# Iterative embedding and reweighting of complex networks reveals community structure

**DOI:** 10.1038/s41598-024-68152-w

**Published:** 2024-07-26

**Authors:** Bianka Kovács, Sadamori Kojaku, Gergely Palla, Santo Fortunato

**Affiliations:** 1 Department of Biological Physics, Eötvös Loránd University, Budapest, Pázmány P. stny. 1/A, 1117 Hungary; 2 Luddy School of Informatics, Computing, and Engineering, Indiana University, 1015 East 11th Street, Bloomington, IN 47408 USA; 3 Department of Systems Science and Industrial Engineering, SUNY Binghamton, P.O. Box 6000, Binghamton, NY 13902 USA; 4 Health Services Management Training Centre, Semmelweis University, Budapest, Kútvölgyi út 2., 1125 Hungary

**Keywords:** Complex networks, Statistical physics

## Abstract

Graph embeddings learn the structure of networks and represent it in low-dimensional vector spaces. Community structure is one of the features that are recognized and reproduced by embeddings. We show that an iterative procedure, in which a graph is repeatedly embedded and its links are reweighted based on the geometric proximity between the nodes, reinforces intra-community links and weakens inter-community links, making the clusters of the initial network more visible and more easily detectable. The geometric separation between the communities can become so strong that even a very simple parsing of the links may recover the communities as isolated components with surprisingly high precision. Furthermore, when used as a pre-processing step, our embedding and reweighting procedure can improve the performance of traditional community detection algorithms.

## Introduction

Recent advances in machine learning have opened new productive research directions in the study of networks (or graphs). Graph embeddings are paradigmatic examples. They represent the structure of a graph via the geometric relations of a set of points arranged in a low-dimensional vector space, where the points are the network nodes and some features of the original network are preserved. Once the graph has been embedded, one can operate on the resulting spatial distribution of points by using the wealth of tools that are available in continuous metric spaces, in particular the possibility of computing distances between the points.

Graph embeddings have been instrumental in various graph data applications, including link prediction^1–4^, node classification^5–9^, and community detection^10–31^. Community detection is a pivotal task in network analysis because communities play key roles in the dynamics and functionality of networks^32–34^. Communities are groups of nodes with a significant density of internal links, whereas the density of links connecting the groups to each other is comparatively lower. Since graph embedding methods typically place closely connected nodes in a network at nearby points in the embedding space, prominent communities are often embedded as compact, well-separated clusters^13,35^. These clusters can then be identified using data clustering techniques such as *k*-means clustering^36^ or DBSCAN^37^. Alternatively, the node proximity in the embedding can be used to facilitate network community detection algorithms by generating a good initial partition^21^ or defining link weights^19^. Whether it is used for data clustering or enhancing network community detection algorithms, the applicability of graph embedding for the identification of communities depends on the ability of the embedding to project communities into distinct, compact clusters. This can be challenging, particularly when different communities are connected by many links. However, even if communities are not well separated in the network, embeddings can still capture node proximities, tending to place nodes within the same community closer together. This proximity information can be leveraged to refine the embedding, resulting in better-defined, compact community clusters that can be more easily identified using data clustering techniques.

We propose an iterative procedure, called *Iterative Embedding and ReWeighting* (IERW), consisting of embedding the network and reweighting its links until a stable weighted graph configuration is reached. We find that, by utilizing information about node proximities derived from the embedding, we can obtain weighted networks in which the communities of the original graph are more and more pronounced over the iterations and easier to find. This effect can be so strong that it allows the recovery of communities by simply removing the longest links of the final weighted graph and identifying the connected components of the resulting network. This simple method is competitive with traditional community detection methods on synthetic graphs generated by the planted partition (PP) model^38^ and can outperform them on the more realistic Lancichinetti–Fortunato–Radicchi (LFR) benchmark^39^. Delivering link weights that strengthen the communities of the original network, IERW can also improve the performance of traditional community detection methods like Louvain^40^, Infomap^41^ or label propagation^42^. In addition, tests on real networks also show the benefits of applying IERW as a pre-processing step in terms of the increased similarity between the ground truth partitioning and the modules detected by the aforementioned traditional community finding methods.

Formerly, an iterative embedding method has been proposed in the field of graph neural networks, where both the graph structure and the embedding are learned in an iterative manner, aiming for a better representation^43^. In parallel, an iteration of node2vec embedding^8^ using *k*-means clustering^36^ cost regularization has been also proposed^44^, whereas in an alternative approach, specifically tailored for hyperbolic embedding based on the random hyperbolic graph^45,46^, the model likelihood was regularized iteratively by taking into account also the communities^47^. Our work provides a more general framework, allowing the inclusion of any embedding method in general. In the present study, we apply both Euclidean and hyperbolic embedding algorithms, all leading to similar results at the qualitative level.

## Results

### Iterative embedding and reweighting

**Figure 1:**
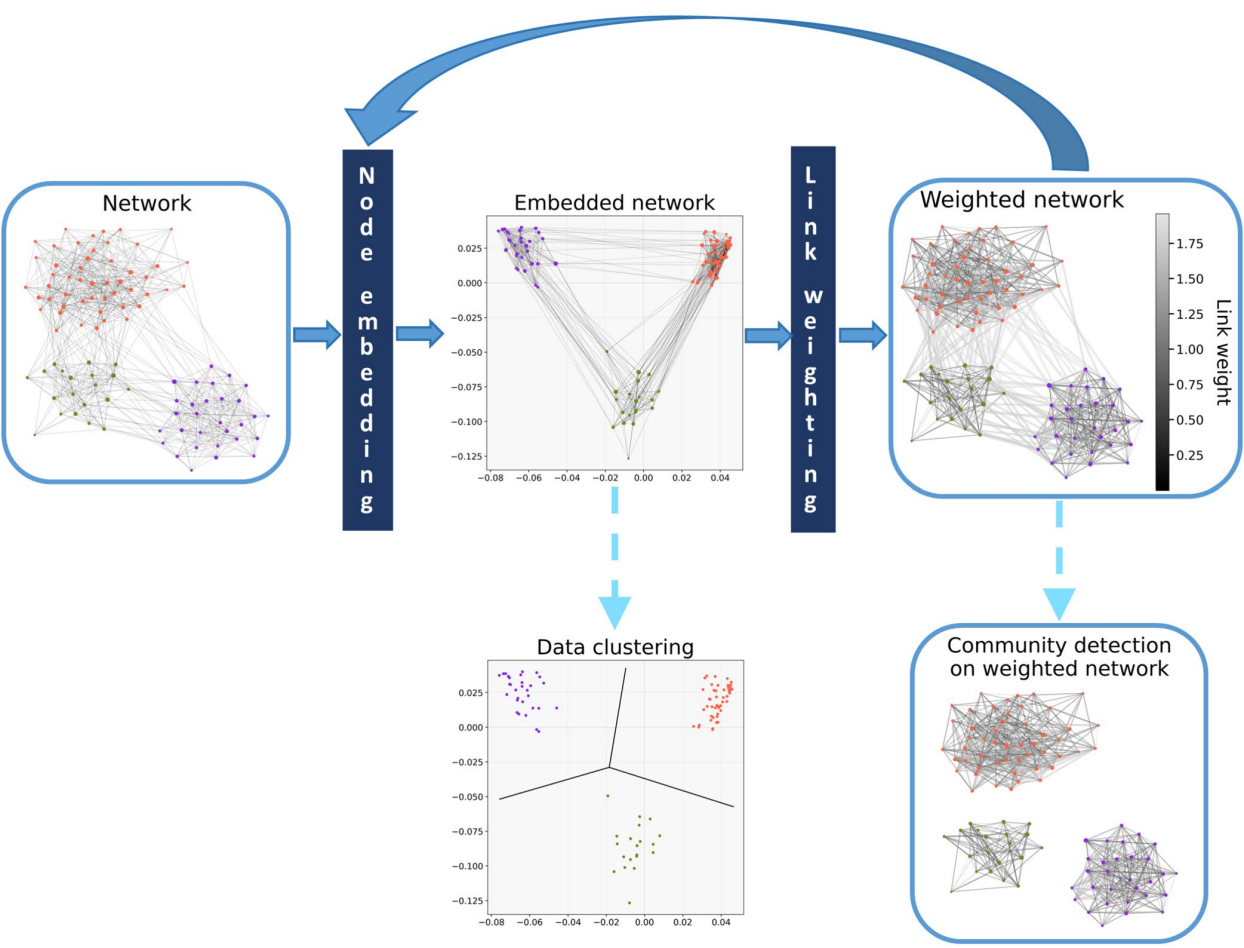
Flowchart of the Iterative Embedding and ReWeighting process. IERW embeds a network into a vector space, where nodes belonging to the same communities are closer to each other compared to nodes from different communities. Then, IERW generates a weighted network with the same sets of nodes and edges, where the edge weights reflect the angular relations of the network nodes in the embedding space. Repeating these two steps, IERW iteratively embeds a weighted network and reweights its links until the variation in the average edge weight within one iteration falls below a specified threshold. Finally, the communities can be identified using data clustering or network community detection algorithms. The example network was generated by the stochastic block model and embedded with Laplacian Eigenmaps on the Euclidean plane. The coloring of the nodes indicates the block memberships assigned by the stochastic block model.

Given an embedding that can form dense spatial clusters from nodes that are strongly connected to each other, it can be expected that when the cohesiveness within the network communities and the separation between them are enhanced via some link weights, then a repeated embedding can further increase the density of the initial spatial clusters. Following this concept, as it is shown in Fig. 1, the proposed Iterative Embedding and ReWeighting (IERW) process repeatedly arranges the network nodes in a vector space according to the topological relations between them and assigns weights to the links of the network in accordance with the geometric relations between the nodes in the previous embedding. During this process, no new links are introduced, only the existing links are reweighted. This framework provides two opportunities for community detection: one can either use standard data clustering methods on the spatial node arrangements generated by the embedding steps, or utilize both the network topology and the geometric relations between the nodes by applying a community detection method on the weighted networks obtained from the link weighting steps.

While our IERW framework is agnostic to the method applied for network embedding, we illustrate the effectiveness of the iterative embedding by focusing on four embedding algorithms (described in the Methods section): Laplacian Eigenmaps (LE)^48^, TRansformation of EXponential shortest Path lengths to hyperbolIC measures (TREXPIC)^49^, Isomap (ISO)^50^ and node2vec^8^. All the applied embedding methods are capable of embedding connected, possibly weighted undirected networks without self-loops and parallel edges in either Euclidean (LE, ISO, node2vec) or hyperbolic (TREXPIC) spaces of any number of dimensions *d*. While LE, TREXPIC and ISO are dimensional reduction techniques based on matrix factorization, in node2vec a neural network creates embeddings based on random walks performed along the network.

As it is detailed in the Methods section, two of the considered methods, namely LE and TREXPIC build on relatively fast-changing, exponential measures of the topological proximity and distance between the network nodes. Following this idea, in order to emphasize the differences between the connectedness of different node pairs in the case of ISO as well, we created a modified version of this embedding method by inserting an exponentialization step into the algorithm. Similarly, we also included exponentialization in the iteration of node2vec, where we left the embedding algorithm itself unaltered but chose an exponential link weight function in IERW. The positive effect of introducing exponentialization in ISO and node2vec is demonstrated in Sect. S2 of the Supplementary Information. In all figures appearing in this paper, we utilised the exponentialization in both ISO and node2vec. Note that the exponentialization step has a tunable constant $t>0$ in the case of all four embedding methods. We did not search for its optimal value in each task individually but used the default setting in all of our measurements. Therefore, our results achieved with IERW may not be the best possible outcomes and there may be room for improvement. The effect of changing *t* in the exponentialization step of the different embedding methods is examined in Sect. S3 of the Supplementary Information.

A crucial step of IERW is the calculation of the link weights based on the positions of the connected nodes in the previous embedding. It is important to bear in mind that the different embedding methods may need different types of link weights as input. Traditionally, in network science link weights represent the intensity or strength of the connection, where a high weight value refers to a strong, close relation between the given node pair. However, some of the embedding methods originate from algorithms initially designed to provide low-dimensional approximations of distances in high-dimensional point clouds, where a high value associated to a node pair refers to a high distance and therefore, presumably a weak connection or a distant relation. Among the embedding methods used in this paper, LE, TREXPIC and ISO expect such distance-like link weights when encountering a weighted link list as an input. In contrast, node2vec expects proximity-like link weights, matching the traditional weight definition in network science.

Although optimizing for different geometric measures on the level of pairwise node-node relations (see the Methods section and Sect. S1 of the Supplementary Information for the details), all the four examined embedding methods tend to place the nodes within the same communities at rather similar angular coordinates, i.e. at small angular distances $\Delta \theta$ from each other. For this reason, to make the embeddings gradually more focused on the community structure, we always defined the link weights in IERW based on the angular relations between the connected nodes. Since cosine distance and cosine proximity are both well-known measures of the angular relations of network nodes, we built our link weighting formulas in IERW on $\cos (\Delta \theta )$. This can be easily calculated for the *d*-dimensional Cartesian position vectors ${\underline{y}}_i$ and ${\underline{y}}_j$ of nodes *i* and *j* in both the Euclidean and the hyperbolic embedding space as

1 $$\begin{aligned} \cos (\Delta \theta _{ij})=\frac{{\underline{y}}_i\cdot {\underline{y}}_j}{\Vert {\underline{y}}_i\Vert \,\Vert {\underline{y}}_j\Vert } \end{aligned}$$

from the dot product ${\underline{y}}_i\cdot {\underline{y}}_j=\sum _{\ell =1}^d{\underline{y}}_i(\ell )\,{\underline{y}}_j(\ell )$ and the Euclidean norms $\Vert {\underline{y}}_i\Vert =\sqrt{\sum _{\ell =1}^d {\underline{y}}_i(\ell )^2}$ and $\Vert {\underline{y}}_j\Vert =\sqrt{\sum _{\ell =1}^d {\underline{y}}_j(\ell )^2}$. The exact definition of the link weighting formula applied in IERW is given in the Methods section for each embedding method.

Besides the link weights, we also have to specify the number of dimensions *d* of the embedding space and a stopping criterion for the iteration to make the IERW framework completed. In the case of the matrix factorization methods (LE, TREXPIC and ISO), we aimed for an embedding dimension *d* equal to $d=C-1$, where *C* denotes the supposed number of communities in the network, which we determined from the eigengap of a normalized graph Laplacian (see Methods). Node2vec, however, often works better with a large *d* in practice due to the nature of the training algorithm. More specifically, node2vec is trained with the stochastic gradient descent algorithm, which regularizes node2vec and prevents it from overfitting^51^. For this reason, we simply used node2vec with a fixed value of $d=64$, corresponding to one of the standard choices in the literature. According to our measurements presented in Sect. S3.3 of the Supplementary Information, while LE, TREXPIC and ISO indeed seem to require a rather specific number of embedding dimensions, the performance of node2vec shows comparatively weak dependence on the value of *d*.

Finally, the stopping criterion for IERW was based on monitoring the relative change in the average link weight ${\bar{w}}$ between subsequent iterations, and the process was terminated when this quantity dropped below a certain threshold, namely when we reached

2 $$\begin{aligned} \frac{|{\bar{w}}_{\textrm{current}}-{\bar{w}}_{\textrm{previous}} |}{{\bar{w}}_{\textrm{current}}}\le 0.001. \end{aligned}$$

Note that we stopped the iteration process after the 20th iteration even if the stopping criterion in Eq. (2) has not been fulfilled yet.

**Figure 2:**
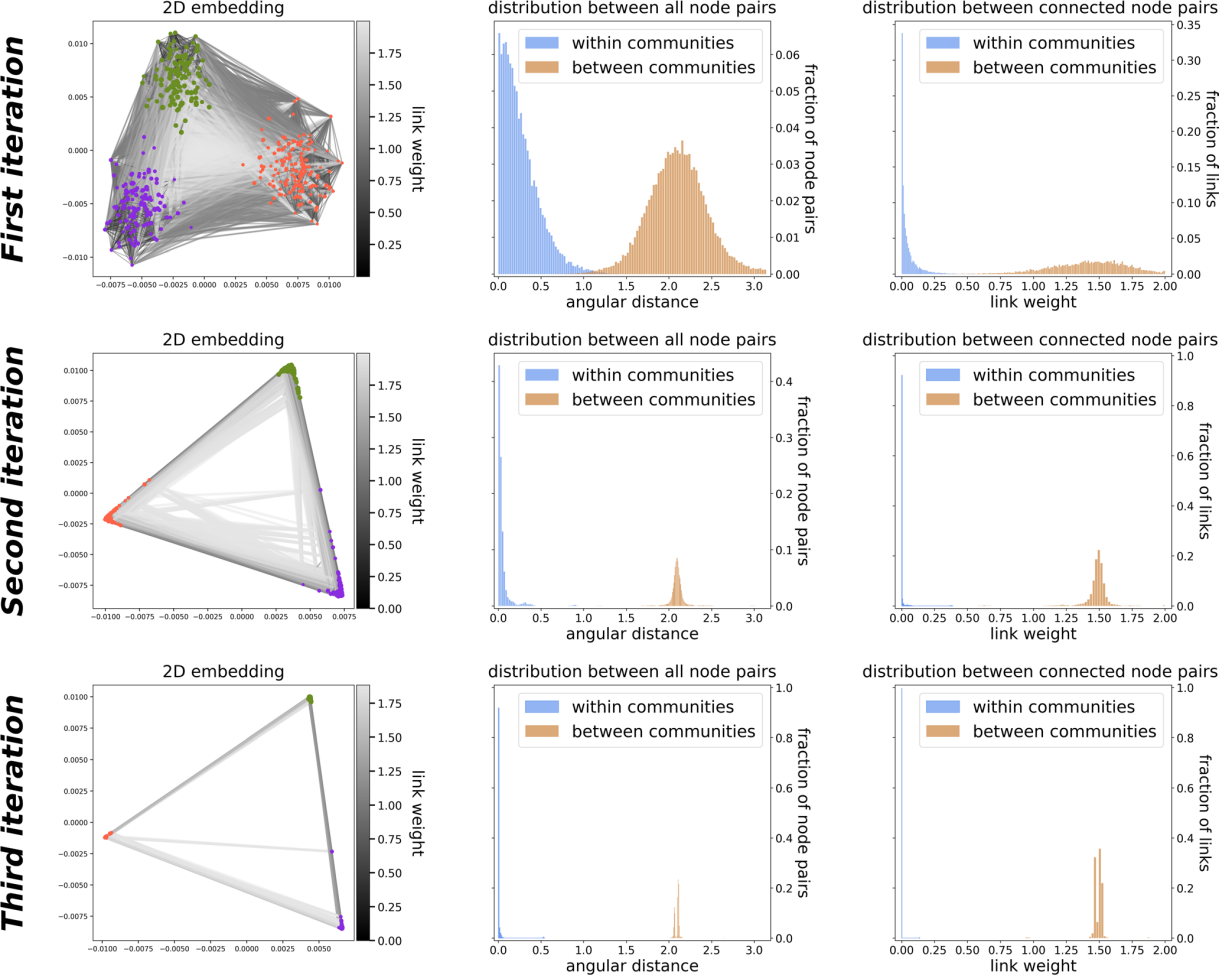
Example of IERW. A network with three communities built by the stochastic block model was embedded three times in the 2-dimensional Euclidean space with Laplacian Eigenmaps. Each row of panels corresponds to one iteration. Initially, all the link weights were 1, and we updated the weights after each embedding using the angular distances $\Delta \theta _{ij}$ as $w_{ij}=1-\cos (\Delta \theta _{ij})$. The left column of panels shows the embeddings, denoting the smaller link weights (that indicate smaller angular distances, and thus, stronger connections) at the end of the given iteration with darker and narrower lines, and coloring the network nodes according to the planted blocks. The column in the middle shows the distribution of the angular distances between all the node pairs in the embedding of the given iteration, while the right column shows the distribution of the link weights of the network.

To demonstrate how IERW works, Fig. 2 shows three iterations using LE, performed on a network generated by the stochastic block model (SBM)^52^ with three communities of size $|\mathcal {A}|=150$ (orange), $|\mathcal {B}|=130$ (purple) and ${|\mathcal {C}|=120}$ (green). In the SBM, the link probability between two nodes only depends on their respective memberships. For three communities these probabilities thus fill a $3\times 3$ stochastic block matrix ${{\varvec{M}}}$, which in our case is3According to Fig. 2, IERW turns the communities into more and more concentrated spatial clusters. Consequently, the distribution of the angular distances between all the node pairs (middle column) and also between the connected node pairs (right column) split into two peaks each with increasing separation. One peak corresponds to the node pairs of the same community (blue) whereas the other refers to the node pairs in different communities (orange).

### Angular separation of communities in iteratively embedded networks

We applied IERW to synthetic networks generated by the planted partition (PP) model^38^ or the Lancichinetti–Fortunato–Radicchi (LFR) model^39^. A key advantage of these generative models is that they enable the definition of communities with tunable internal and external link densities, allowing to control the difficulty of the network clustering problem through the adjustment of the mixing parameter $\mu$, which corresponds to the average fraction of neighbors of one node belonging to communities different from the one of the node. Details on the synthetic network generation are provided in the Methods section.

**Figure 3:**
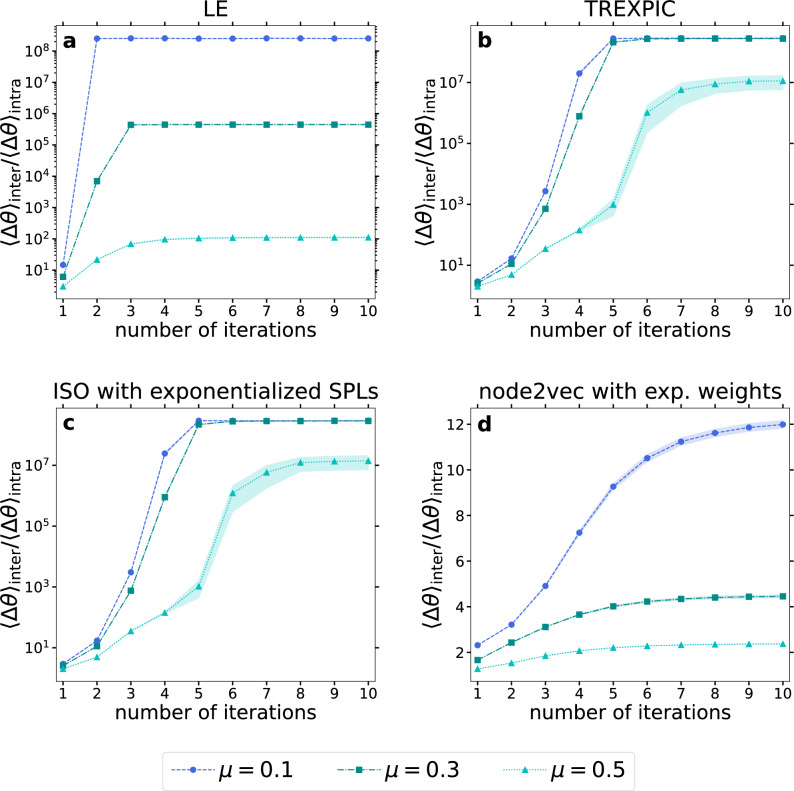
IERW increases angular separation of communities in networks generated by the PP model. We plot the ratio between the average angular distance of all possible node pairs in different communities and in the same community as a function of the number of IERW iterations for LE (**a**), TREXPIC (**b**), ISO with exponentialized shortest path lengths (**c**) and node2vec (**d**). Curves of different colors correspond to different values of the mixing parameter $\mu$. Each depicted data point was obtained by averaging the results over 100 different network realizations, and the shaded areas show the standard error of the mean.

In Fig. 3, we show the ratio between the average inter-community angular distance $\langle \Delta \theta \rangle _{\textrm{inter}}$ (i.e., the average of the angular distances over all the node pairs of different communities) and the average intra-community angular distance $\langle \Delta \theta \rangle _{\textrm{intra}}$ (i.e., the average of the angular distances over all the node pairs belonging to the same community) as a function of the number of IERW iterations performed for networks generated by the PP model. According to the figure, the $\langle \Delta \theta \rangle _{\textrm{inter}}/\langle \Delta \theta \rangle _{\textrm{intra}}$ ratio starts to increase over the iterations and then saturates for all the four studied embedding methods, reaching in some cases extremely high values, which indicates a strong separation between the planted communities in the embedding space. Naturally, when the mixing parameter $\mu$ is only 0.1, the angular separation ratio $\langle \Delta \theta \rangle _{\textrm{inter}}/\langle \Delta \theta \rangle _{\textrm{intra}}$ is higher compared to the case of moderate mixing between the communities at $\mu =0.3$, that in turn surpasses in every iteration the results observed for the relatively strong mixing of $\mu =0.5$, where nodes have roughly the same number of internal and external neighbors. Nevertheless, the curves of the angular separation ratio are increasing as a function of the number of iterations even at $\mu =0.5$, indicating that our iterative embedding framework helps in separating the planted communities in the embedding space.

**Figure 4:**
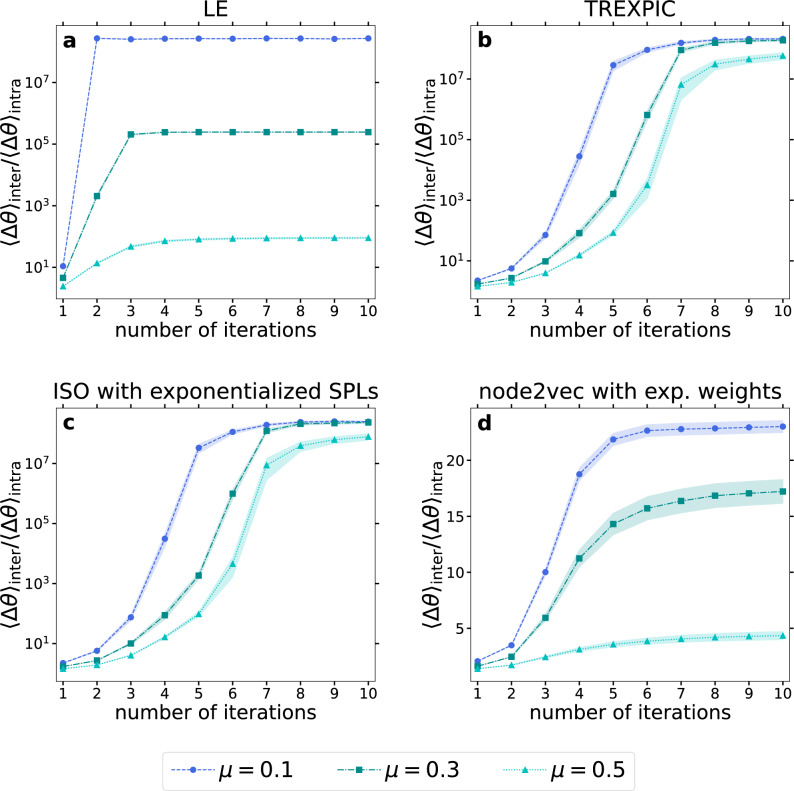
IERW increases angular separation of communities in networks generated by the LFR benchmark. We plot the ratio between the average angular distance of all possible node pairs in different communities and in the same community as a function of the number of IERW iterations for LE (**a**), TREXPIC (**b**), ISO with exponentialized shortest path lengths (**c**) and node2vec (**d**). Curves of different colors correspond to different values of the mixing parameter $\mu$. Each depicted data point was obtained by averaging the results over 100 different network realizations, and the shaded areas show the standard error of the mean.

In Fig. 4, we display the results for the angular separation of planted communities in LFR networks. The qualitative behaviour of the $\langle \Delta \theta \rangle _{\textrm{inter}}/\langle \Delta \theta \rangle _{\textrm{intra}}$ ratio is quite similar to that in Fig. 3: the angular separation ratio starts with an increasing trend and then saturates as a function of the number of IERW iterations. The lower the $\mu$ value, the higher the saturated ratio. As in Fig. 3, the actual value of the angular separation ratio can grow even above $\langle \Delta \theta \rangle _{\textrm{inter}}/\langle \Delta \theta \rangle _{\textrm{intra}}=10^7$.

### Separation of communities via weight thresholding

To give further perspective on the communities’ strong separation resulting from our framework, here we show that even a clearly sub-optimal, overly simplistic community extraction method can provide surprisingly good results when exploiting the geometric information encoded in the link weights at the end of the IERW process. The basic idea is to set a threshold aiming to separate the links that connect members of the same community from those between nodes of different communities. By deleting the links on one side of the threshold—those that are suspected to connect different communities—, the network falls apart into disconnected components that we may identify as the detected communities. Since, as it is illustrated by Fig. 2, a rather large gap can emerge between the weight of intra- and inter-community links during IERW, this simple weight thresholding strategy (detailed in the Methods section) can actually work effectively under optimal circumstances. Note that the iteration of the embedding is indeed necessary to make the weight thresholding work as the weight thresholding after a single embedding yields poor community detection performance (see Sect. S4 of the Supplementary Information).

**Figure 5:**
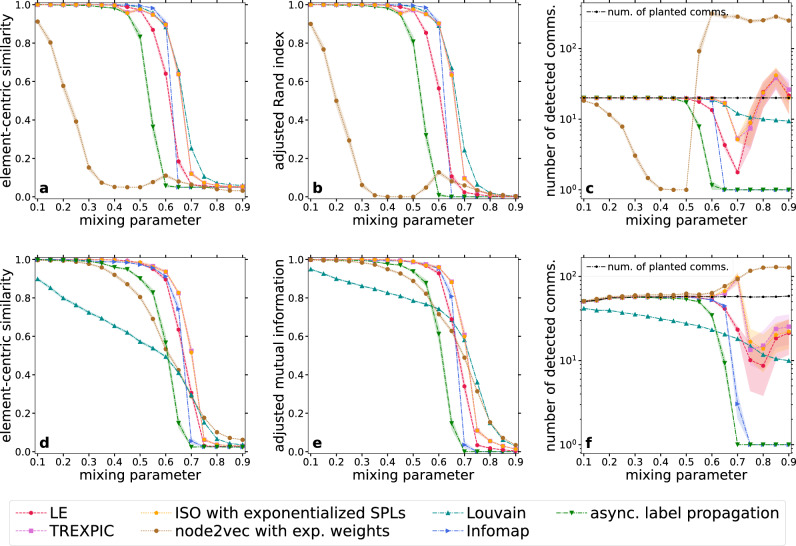
Extracting communities via weight thresholding the network yielded by IERW. Panels (**a**), (**b**) and (**c**) refer to input networks generated by the PP model, while panels (**d**), (**e**) and (**f**) deal with input networks obtained from the LFR benchmark. As a reference, the three dash-dotted lines show the results achieved by traditional network community detection methods on the initial unweighted graphs: Louvain (dark cyan upward-pointing triangles), Infomap (blue right-pointing triangles) and asynchronous label propagation (green downward-pointing triangles). The other four colored lines illustrate the results for a simple weight thresholding that we applied on the final weighted networks obtained from IERW with LE (red hexagons), TREXPIC (purple squares), ISO with exponentialized shortest path lengths (orange pentagons) and node2vec with exponentialized link weights (brown circles). We performed the community detection with all the methods only once for each network. Each displayed data point corresponds to a result averaged over 100 networks, and the error bars indicate the standard error of the mean.

Although the applied weight thresholding approach is rather crude, thanks to the large angular separation that IERW achieves between the communities, it can still yield results comparable in quality to state-of-the-art community-finding methods. In Fig. 5, we compare the performance of the weight thresholding with that of three commonly used, well-established network community detection methods. Even though all three methods are able to take into account link weights, in the case of Fig. 5 we applied them on the original, unweighted test graphs and not on the weighted versions obtained from the IERW process.

First, we used the Louvain algorithm^40,53^, performing a heuristic maximization of the well-known modularity by Newman and Girvan^54,55^, which compares the observed internal link density of the communities to its expected value. Though Louvain can unfold a hierarchical community structure (with nested modules and submodules), we always considered the top-level community structure, i.e. the one with the highest modularity.

Besides, we applied the Infomap algorithm^41,56^, which relies on a heuristic minimization of the so-called map equation^41^. It assumes that communities are regions of a network within which random walkers spend a relatively long time, and searches for the community structure that is the best for compressing the description (i.e., the code length) of random walk trajectories along the network. Infomap, just like Louvain, can create a hierarchy of network partitions; here we considered the lowest hierarchical level, yielding the shortest description length.

Finally, we used the asynchronous label propagation algorithm^42,57^, which does not aim at the optimization of any predefined measure but simulates the diffusion of the nodes’ community labels along the links, regularly updating the community membership of each node following the current majority of the neighboring nodes, expecting that eventually a consensus on a unique label becomes established within densely connected groups of network nodes. Following the suggestion in Ref.^42^, we completed the label propagation process by separating groups of nodes that ended up with the same label but were not connected to each other.

The PP graphs (top row of panels) and the LFR networks (bottom row of panels) studied in Fig. 5 are the same as in Figs. 3 and 4, respectively. The network generation process is detailed in the Methods section. To evaluate the performance of the examined community detection methods, we measured the number of detected communities (right column of Fig. 5), as well as different similarity scores (left and middle columns of Fig. 5) between the planted and the detected community structures.

First, we calculated the element-centric similarity (ECS)^58,59^ between the detected and planted partitions (Fig. 5a,d), which is a measure comparing node-node transition probabilities in random walks performed along the two graphs of cluster-induced (i.e., groupmate) relationships derived from the two partitions. ECS has its maximum of 1 for identical partitions and decreases as the similarity between the compared divisions declines. Note that the expected value of ECS when inputting two random partitions having an equal number of groups and equal group sizes is not set to 0^27^. Furthermore, the only tunable parameter of the method for non-hierarchical clusterings is given by the restart probability of the random walks, but it does not have any effect in the case of hard partitions^27^, so in our measurements we simply used its default value.

Besides the ECS, following the suggestions of Ref.^60^, we used the adjusted Rand index (ARI)^61–64^ for the PP networks (Fig. 5b), where the group sizes in the ground truth clustering were equal, and the adjusted mutual information (AMI)^65–68^ for the LFR networks (Fig. 5e), where the ground truth partition was unbalanced with respect to the group sizes, i.e. strongly different community sizes occurred. Both ARI and AMI take the value of 1 in the case of perfect agreement between two partitions, and (being corrected or adjusted for the agreement emerging only by chance) the value of 0 on expectation when comparing random partitions having the same number of communities and the same community sizes. ARI and AMI can decrease even below 0 if the considered two clusterings differ to a large extent. While ARI is a pair-counting similarity measure that relies on the number of node pairs being groupmates or belonging to different groups in both the planted and the detected community structures, AMI is an information-theoretic quantity operating with the community membership probabilities of a randomly chosen node, which are calculated based on the relative size of the communities and the overlaps between the groups from the different partitions. Though there are several different possibilities for the normalization in the AMI formula, we always normalized with the maximum of the Shannon entropies associated with the two partitions to be compared.

In the case of the PP model, the community-finding performance of the weight thresholding based on iterated node2vec is poor according to both ECS (Fig. 5a) and ARI (Fig. 5b). In the meantime, the similarity scores achieved using IERW in the case of TREXPIC or ISO with exponentialized shortest path lengths are very close to that of Infomap and Louvain in Fig. 5a,b. The results based on iterated LE fall slightly behind, although they still surpass the scores of asynchronous label propagation.

In the case of the LFR benchmark, the results for the weight thresholding based on IERW using both TREXPIC and ISO with exponentialized shortest path lengths slightly exceed that of even Infomap (Fig. 5d,e), which is followed closely by the results achieved using iterated LE. Asynchronous label propagation falls somewhat behind similarly to the PP case, but here it is followed relatively closely by the results based on iterated node2vec, which in turn surpasses Louvain. Louvain has a poor performance on LFR graphs due to the resolution limit of modularity maximization^69^.

### Facilitating traditional community detection methods with iterative embedding

**Figure 6:**
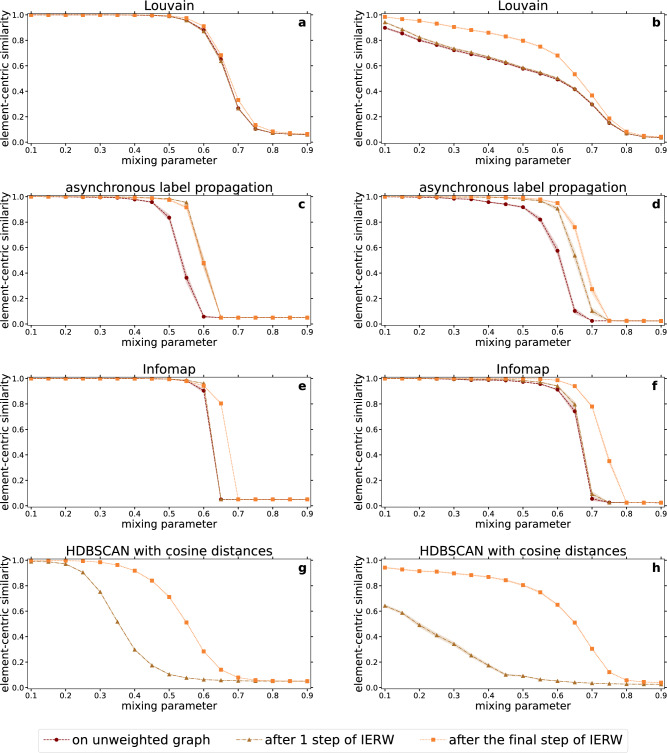
Performance of standard clustering methods on the weighted networks and the embeddings derived by IERW using node2vec with exponentialized link weights. Each row of panels corresponds to a different community detection method. The left column refers to networks generated by the PP model and the right one to networks generated by the LFR benchmark. We performed the community detection with all the methods only once for each network. Each displayed data point corresponds to a result averaged over 100 networks, and the error bars depict the standard error of the mean.

As it is shown in Fig. 1, our IERW process can aid community detection in two different ways: one may either apply standard data clustering techniques on the spatial node arrangements obtained from the embedding steps, or opt for community-finding methods developed for weighted networks, taking into account both the network topology and the geometric relations of the embedded nodes. In Fig. 6, we show examples for both options. On the one hand, we compare the performance of traditional network community-finding approaches on unweighted synthetic benchmark graphs to the results achieved when these methods are augmented by the link weights obtained from a single and multiple iterations of IERW using node2vec. As the network community detection methods, we employed Louvain^40,53^ (Fig. 6a,b), asynchronous label propagation^42,57^ (Fig. 6c,d) and Infomap^41,56^ (Fig. 6e,f). In addition, we tested Hierarchical Density-Based Spatial Clustering of Applications with Noise (HDBSCAN)^70–72^ on both the first and the iterated node embeddings (Fig. 6g,h), inputting only the cosine distance between all the possible node pairs in the embedding space. The left column of Fig. 6 displays the ECS scores achieved for the PP test graphs of Figs. 3 and 5a–c, whereas the right column of Fig. 6 refers to the LFR networks examined in Figs. 4 and 5d–f. We repeated the experiments shown in Fig. 6 using LE, ISO and TREXPIC embeddings too: the results, qualitatively very similar, are shown in Sect. S5 of the Supplementary Information.

Regarding traditional network community detection methods, it is important to keep in mind that while Louvain, asynchronous label propagation and Infomap expect proximity-like link weights, the link weights $w_{ij}$ provided by IERW can be both distance-like (when using LE, ISO and TREXPIC) and proximity-like (in the case of node2vec). Hence, following a similar practice to the one suggested in Ref.^19^, in Sect. S5 of the Supplementary Information we used a conversion formula

4 $$\begin{aligned} {\tilde{w}}_{ij}=\frac{1}{w_0+w_{ij}} \end{aligned}$$

on the link weights obtained from IERW with LE, ISO and TREXPIC before applying Louvain, asynchronous label propagation or Infomap, where $w_{0}>0$ is a tunable parameter. In general, by choosing a small $w_0$ we put more emphasis on the distances close to 0, in agreement with the expectation that the distances within communities eventually decrease over the iterations. Our analysis detailed in Sect. S5 of the Supplementary Information shows that $w_0$ can affect the performance of the network community-finding methods when using IERW with LE, ISO and TREXPIC. Similarly, we also used a conversion formula

5 $$\begin{aligned} {\tilde{w}}_{ij}=w_0+w_{ij} \end{aligned}$$

after applying IERW with node2vec, setting $w_0$ to 1.0 in Fig. 6a–f, as we found that this shifting of all the proximity-like exponential link weights provided by IERW can improve the performance of all the examined traditional network community detection methods.

As it can be seen in Fig. 6, the node2vec-based IERW process can strongly improve the performance of standard clustering methods. We observed the largest improvement in the case of Louvain, when applied to LFR networks (Fig. 6b). It is well-known that community-finding methods based on modularity maximization (such as Louvain) may fail in detecting small communities^69^. Since the size distribution of the communities is relatively broad in the examined LFR networks, the ECS achieved on the original unweighted test graph (dark red curve) remains well below 1 already at low $\mu$ values in Fig. 6b, indicating that Louvain in itself cannot fully uncover the planted community structure. The performance after only a single embedding (light brown curve) is similar to what is achieved in the unweighted case. However, when switching to the weighted networks provided by the complete process of IERW (orange curve), the performance greatly improves. Note that in the similar measurements performed with LE, TREXPIC and ISO in Figs. S11–S13 of the Supplementary Information, IERW seems to actually eliminate the resolution limit of modularity optimization, increasing the ECS of Louvain to 1 in a wide range of the mixing parameter.

In the case of Louvain applied to PP networks (Fig. 6a) and Infomap (Fig. 6e,f), the results on the original, unweighted input graphs are already of very high quality. However, a slight increase can still be observed here when switching to the networks weighted by IERW with node2vec. In the case of asynchronous label propagation (Fig. 6c,d), the performance of a single embedding is similar to that of the iterated embedding, both being significantly better compared to the unweighted case. Finally, when applying HDBSCAN to the spatial node arrangements created by node2vec (Fig. 6g,h), although the performance after a single embedding is modest, the iteration of the embedding yields major improvements for both the PP and the LFR graphs.

### Experiments on real-world networks

**Figure 7:**
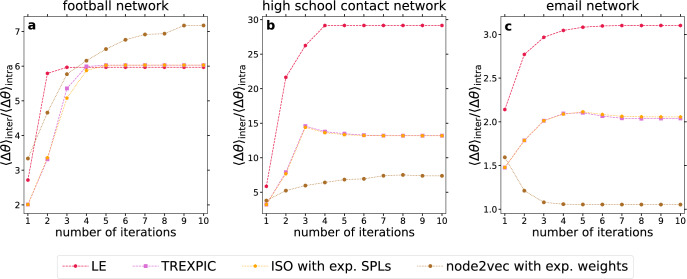
Angular separation of communities during IERW on three real graphs with known community structure. The different panels correspond to different real datasets: panel (**a**) to the American College Football network, panel (**b**) to the contact network of high school students, while panel (**c**) to the email network. Each panel depicts the ratio between the average angular distance of all possible node pairs in different communities and in the same community as a function of the number of IERW iterations for LE (red hexagons), TREXPIC (purple squares), ISO with exponentialized shortest path lengths (orange pentagons) and node2vec with exponentialized link weights (brown circles).

While the previous subsections demonstrate the applicability of IERW on synthetic networks of different levels of mixing between the communities, there is a natural need for the study of real graphs too, as these may not have as clearly defined or regular communities as those produced by network models. Therefore, Fig. 7 and Table 1 show some results for three real-world networks. The first one (Fig. 7a) is the American College Football network^73,74^ with 115 teams as nodes and 613 games as edges, where the 12 ground truth communities are given by the conferences of the football teams. The second real dataset (Fig. 7b) is a network of 2026 contacts of at least 2 minutes length between 227 high school students (see the cumulative contact network for day 2 in Ref.^75^), where a community information describing 10 classes is given. The third real network (Fig. 7c) is based on 16064 emails sent between 986 members of a research institute^76–78^, where the 42 known groups of the nodes correspond to the departments of the institute.

According to Fig. 7, albeit the changes are not as extreme as in the PP graphs (Fig. 3) or the LFR networks (Fig. 4), the increasing tendency of the angular separation between the communities is a common trait of iterated embeddings when applied on real graphs too. The single exception is when we applied IERW with node2vec on the email network (Fig. 7c, brown curve). However, even in this case, the performance of traditional community finding methods may be still better on the iteratively reweighted network than on the original one, as it is shown in Table 1 for Infomap. Furthermore, as it is indicated by Table 1, the communities can be extracted from the weighted versions of the real networks produced by IERW even with a simple weight thresholding (which was tested on synthetic graphs in Fig. 5) with a relatively good accuracy, and the performance of traditional community detection methods (studied on synthetic networks in Fig. 6) was also generally improved by IERW on all the examined real networks.

**Table 1:** Element-centric similarities achieved by different community detection approaches on three real graphs with known community structure.

Community detection method	Football network	High school contact network	Email network
Louvain on unweighted graph	0.786	0.714	0.333
Infomap on unweighted graph	0.826	0.879	0.364
async. label prop. on unweighted graph	0.775	0.777	0.047
weight threshold after IERW with LE	0.819	0.962	**0.484**
weight threshold after IERW with TREXPIC	0.819	0.918	0.393
weight threshold after IERW with ISO	0.819	0.918	0.398
weight threshold after IERW with node2vec	0.755	0.879	0.118
Louvain after IERW with LE	0.819	**1.0**	0.483
Louvain after IERW with TREXPIC	0.819	0.936	0.429
Louvain after IERW with ISO	0.819	0.936	0.438
Louvain after IERW with node2vec	0.827	0.799	0.330
Infomap after IERW with LE	**0.867**	0.879	0.368
Infomap after IERW with TREXPIC	**0.867**	0.879	0.387
Infomap after IERW with ISO	**0.867**	0.879	0.383
Infomap after IERW with node2vec	0.860	0.984	0.418
async. label prop. after IERW with LE	0.819	0.962	0.248
async. label prop. after IERW with TREXPIC	0.819	0.924	0.385
async. label prop. after IERW with ISO	0.819	0.924	0.403
async. label prop. after IERW with node2vec	0.860	0.849	0.047
HDBSCAN after IERW with LE	0.386	**1.0**	**0.484**
HDBSCAN after IERW with TREXPIC	0.386	0.925	0.393
HDBSCAN after IERW with ISO	0.386	0.925	0.369
HDBSCAN after IERW with node2vec	0.479	0.946	0.068

As a reference, the first three rows show the similarity scores achieved by traditional community detection methods on the original, unweighted real graphs. Below that, the performance of weight thresholding, Louvain, Infomap and asynchronous label propagation is listed when utilizing the link weights obtained from the final iteration of IERW with LE, TREXPIC, ISO with exponentialized shortest path lengths and node2vec with exponentialized link weights. Lastly, the bottom of the table indicates the quality of the communities found by HDBSCAN on the final embedding provided by IERW using the studied four embedding methods. The community detection was performed with all the methods only once for each network. For all three examined networks, the best results are written in bold.

## Discussion

We have shown that graph embeddings facilitate the identification of communities, by providing distance- or proximity-based weights to the links of the input graph, which makes its community structure topologically more pronounced and more easily detectable. At the same time, embedding graphs with progressively stronger community structures makes communities more apparent also in the embedding space, where they appear as clouds of points that become more and more compact and separated from each other. These observations inspired our IERW framework, which realizes a simple iterative procedure to ease community detection, where the network is repeatedly embedded and reweighted based on the geometric distance between the endpoints of the links. For embedding methods such as node2vec, where a larger link weight is interpreted as the indicator of a stronger and closer connection, as we keep iterating, intra-community link weights get larger and inter-community link weights get smaller. For the other embedding methods studied in the present paper (where the link weights are assumed to be distance-like), IERW acts in the opposite manner, increasing the weight of inter-community links and decreasing the weight of intra-community links over the iterations. Both cases suggest a simple way to find the clusters: removing inter-community links via weight thresholding. Such an approach, albeit elementary, is competitive with state-of-the-art community detection techniques.

We stress that we only reweight the links of the original graph. If we assigned a weighted link to each pair of nodes, whether they are connected or not, the identification of the communities may become easier but at the cost of having a procedure with at least quadratic complexity in the number of nodes *N*. By focusing on the actual links of the input network, instead, the reweighting procedure has linear complexity in the number of links, which is much lower than $N^2$ on sparse networks. The ultimate complexity of the repeated embedding and weighting steps is determined by the running time of the chosen embedding algorithm. In the case of node2vec, for instance, the complexity of IERW would be $\mathcal {O}\left( E+N\cdot d\cdot \omega ^2\right)$ for a network of *N* nodes and *E* edges when using a *d*-dimensional embedding space and $\omega$ window length (empirically measured running times are also presented in Sect. S7 of the Supplementary Information). Here the results are fairly stable as a function of *d*, so one can pick a fixed value (we used $d=64$ in our experiments). For the other examined embeddings, there is a much stronger dependence on the number of embedding dimensions, and identifying a good range may be costly (see Methods).

Our method could be used as a pre-processing step in a community detection pipeline. We find that a single iteration of IERW can already produce a weighted network having stronger communities than the original graph. Applied after IERW, standard community detection techniques generally deliver better results than when they operate on the initial graph. Interestingly, our reweighting strategy provides a way to mitigate the effect of the resolution limit of modularity maximization, significantly improving the performance of such methods on realistic benchmarks.

Finally, we would like to stress that techniques like IERW could help facilitating other tasks, besides community detection. It would be interesting, for instance, to check whether link prediction also becomes easier on the weighted graphs and/or embeddings built by IERW or similar procedures, bearing in mind that different tasks may need different weighting rules and the application of different geometric measures.

## Methods

### Node embedding with Laplacian Eigenmaps

Based on the eigendecomposition of the Laplacian matrix of a neighborhood graph made from the original data set, the Laplacian Eigenmaps (LE) approach was first devised in Ref.^48^ for mapping data points supplied in a high-dimensional space onto a lower dimensional one. When applied to a weighted network, in the first step the assumed distance-like input weights $w_{ij}$ are converted to proximity-like weights using the exponential formula $w_{ij}'(w_{ij})=\exp (-w_{ij}^2/t)$, where, following the implementation created for Ref.^19^, we set the parameter *t* to be equal to the square of the mean of the distance-like weights. Then, from the corresponding adjacency matrix ${{\varvec{A}}}$ and the diagonal matrix $\varvec{\mathcal {D}}$ with $\mathcal {D}_{ii}=\sum _j A_{ij}$, we can obtain the Laplacian matrix as ${{\varvec{L}}}=\varvec{\mathcal {D}}-{{\varvec{A}}}$. The eigenvectors $f_1,f_2,\dots ,f_d$ satisfying the generalized eigenvector problem ${{\varvec{L}}}\cdot {\underline{f}}_{\ell }=\lambda _{\ell }\cdot \varvec{\mathcal {D}}\cdot {\underline{f}}_{\ell }$ with the smallest non-zero eigenvalues ${\lambda _1\le \lambda _2\le \ldots \le \lambda _d}$ naturally define an embedding in the *d*-dimensional Euclidean space, where the $\ell {\textrm{th}}$ coordinate of the $i{\textrm{th}}$ node is given by the $i{\textrm{th}}$ component of $f_{\ell }$, making strongly connected nodes being as close to each other as possible.

The computational complexity of LE is $\mathcal {O}\left( (d+1)\cdot N^2\right)$, where the dominant contribution comes from the eigendecomposition of the $N\times N$-sized graph Laplacian. A fully detailed algorithmic description of LE is provided in Sect. S1 of the Supplementary Information.

In IERW with LE, we defined the distance-like input weights based on the angular distance $\Delta \theta _{ij}$ in the previous embedding as $w_{ij}=1-\cos (\Delta \theta _{ij})$.

### Node embedding with TRansformation of EXponential shortest path lengths to hyperbolIC measures

The TRansformation of EXponential shortest Path lengths to hyperbolIC measures (TREXPIC) method^49^ embeds networks in a *d*-dimensional hyperbolic space, trying to express the topological node-node distances as hyperbolic distances. First, TREXPIC prepares a matrix ${{\varvec{X}}}$ of expected hyperbolic distances based on the shortest path lengths $\textrm{SPL}_{ij}$ measured along the graph, using the exponential formula $X_{ij}=\exp (-t/\textrm{SPL}_{ij})$. Here we set the parameter $t>0$ to the default value defined in Ref.^49^, given by $t=\sqrt{\ln (1.0/0.9999)\cdot \ln (1.0/0.1)}\cdot \textrm{SPL}_{\textrm{max}}$ with $\textrm{SPL}_{\textrm{max}}$ being the maximal shortest path length found in the network. The distance matrix ${{\varvec{X}}}$ is then converted into the matrix $\varvec{\mathcal {L}}$ of expected pairwise Lorentz products, using the formula $\mathcal {L}_{ij}=\cosh (\zeta \cdot X_{ij})$, where we set $\zeta$ simply to 1, and thus, the curvature of the hyperbolic space $K=-\zeta ^2$ to $-1$. Finally, the matrix $\varvec{\mathcal {L}}$ is subjected to singular value decomposition (formulated as $\varvec{\mathcal {L}}={{\varvec{U}}}\cdot {\varvec{\Sigma} }\cdot {{\varvec{V}}}^{\textrm{T}}$): the length of the node position vectors is calculated from the largest singular value $\sigma _1\equiv \Sigma _{11}$ and the corresponding singular vector ${\underline{u}}_1$ (given by the first column of the matrix ${{\varvec{U}}}$), while the direction vectors of the embedded nodes are calculated from the next *d* singular values ($\sigma _2\ge \sigma _3\ge \cdots \ge \sigma _{d+1}$) and the corresponding singular vectors (${\underline{u}}_2,\,{\underline{u}}_3,\,\ldots ,\,{\underline{u}}_{d+1}$).

The computational complexity of TREXPIC for ${d+1<\ln (N)}$ is dominated by the calculation of the $N\times N$-sized shortest path length matrix, yielding $\mathcal {O}\left( \ln (N)\cdot N^2\right)$, while the computational complexity of the truncated singular value decomposition is $\mathcal {O}\left( (d+1)\cdot N^2\right)$. A fully detailed description of the TREXPIC approach is presented in Sect. S1 of the Supplementary Information.

Similarly to the case of LE, in IERW with TREXPIC we defined the distance-like input weights as $w_{ij}=1-\cos (\Delta \theta _{ij})$ based on the previous embedding iteration.

### Node embedding with Isomap

Similarly to LE, the Isomap (ISO) method was originally proposed^50^ for finding a lower-dimensional representation of a high-dimensional data set using a nearest neighbor graph. Aiming at a mapping between the topological node-node distances and the Euclidean distances in the embedding, a matrix ${{\varvec{I}}}$ of expected pairwise inner products is calculated from the shortest path length (SPL) matrix of the graph to be embedded, placing the center of mass of the embedded graph at the origin. In the present paper, we followed the implementation applied in Ref.^19^, which performs not the eigendecomposition but the singular value decomposition of the matrix ${{\varvec{I}}}$. This singular value decomposition (formulated as ${{\varvec{I}}}={{\varvec{U}}}\cdot {\varvec{\Sigma} }\cdot {{\varvec{V}}}^{\textrm{T}}$) provides the node coordinates in the *d*-dimensional Euclidean space: by taking the *d* largest singular values ${\sigma _1\ge \sigma _2\ge \cdots \ge \sigma _d}$ and the corresponding singular vectors ${\underline{u}}_1,\,{\underline{u}}_2,\,\ldots ,\,{\underline{u}}_d$, the $\ell {\textrm{th}}$ component of the position vector of the $i{\textrm{th}}$ network node is defined as ${\underline{y}}_i(\ell )=\sqrt{\sigma _{\ell }}\cdot {\underline{u}}_{\ell }(i)$.

To improve the performance of IERW, we introduced an alternative version of ISO that is built on exponentialized shortest path lengths, similarly to TREXPIC. Here, the original formula $D_{ij}=\textrm{SPL}_{ij}$ of the expected pairwise Euclidean distances is replaced by $D_{ij}=\exp (-t/\textrm{SPL}_{ij})$, where $t>0$ is a tunable parameter. We used the same setting for this *t* parameter as in the default case of TREXPIC, namely ${t=\sqrt{\ln (1.0/0.9999)\cdot \ln (1.0/0.1)}\cdot \textrm{SPL}_{\textrm{max}}}$, where $\textrm{SPL}_{\textrm{max}}$ is the largest shortest path length of the examined network. The beneficial effect of exponentialization in ISO is demonstrated in Sect. S2 of the Supplementary Information.

The computational complexity of ISO for ${d<\ln (N)}$ is dominated by the calculation of the $N\times N$-sized shortest path length matrix, yielding $\mathcal {O}\left( \ln (N)\cdot N^2\right)$, while the computational complexity of the truncated singular value decomposition is $\mathcal {O}\left( d\cdot N^2\right)$. A fully detailed description of ISO embeddings is given in Sect. S1 of the Supplementary Information.

In complete analogy with LE and TREXPIC, in IERW with both versions of ISO we defined the link weights of the network based on the previous embedding iteration simply as $w_{ij}=1-\cos (\Delta \theta _{ij})$.

### Node embedding with node2vec

The node2vec method^8^ provides Euclidean node embeddings based on random walks in the network. The central idea is to use the sequences of the visited nodes as textual input for the word2vec^79^ method, originally designed to embed words from a large text corpus into a vector space. In the present paper, we followed the parameter setting proposed in Ref.^26^ by setting the number of walks started from each node to 80, the length of the random walk to 10 and the length of the considered context windows in word2vec to $\omega =10$. The parameters *p* and *q*, controlling the locality and the depth of the random walks were set to the default value of $p=q=1$.

The computational complexity of creating a *d*-dimensional embedding for a network of *N* nodes and *E* edges with node2vec is $\mathcal {O}\left( E+N\cdot d\cdot \omega ^2\right)$. Note that since node2vec operates with random walks, it is a stochastic embedding method. Nonetheless, as we performed all of our measurements on multiple network samples anyway, we ran IERW with node2vec only once for each network. A more detailed description of the node2vec method is given in Sect. S1 of the Supplementary Information.

When provided with a weighted input network, the random walk transition probabilities are modified in node2vec according to the link weights, where a higher link weight is accompanied by a higher transition probability. According to that, opposite to the previous embedding methods, node2vec expects proximity-like link weights instead of distance-like weights. To utilize the beneficial effects of exponentialization in IERW also with node2vec, here we defined exponential link weights based on the previous embedding iteration as $w_{ij}=\exp \left( t\cdot [\cos (\Delta \theta _{ij})-1]\right)$, where the parameter *t* was set to $t=10\cdot {\bar{\kappa }}/{\hat{\kappa }}$ with ${\bar{\kappa }}$ denoting the average and ${\hat{\kappa }}$ the mode of the node degrees, respectively. The advantage of the exponentialization over the application of a simple proximity-like link weight formula given by $w_{ij}=\cos (\Delta \theta _{ij})+1$ is demonstrated in Sect. S2 of the Supplementary Information.

### Choosing the number of embedding dimensions

When using IERW with node2vec, we followed one of the standard choices in the literature and simply set the number of embedding dimensions *d* always to 64. However, as it is demonstrated in Sect. S3 of the Supplementary Information, the performance of LE, ISO and TREXPIC shows a relatively strong dependence on the setting of *d*, and in the case of these matrix decomposition methods, it seems that the best choice is a *d* close to the number of communities in the examined network. Therefore, before applying IERW with LE, ISO or TREXPIC, we estimated the number of planted communities *C* based on the number of non-zero eigenvalues below the largest eigengap of the normalized Laplacian matrix of the given network, and using this estimation, we set the number of embedding dimensions to $d=C-1$, which fits the expectation that e.g. a two-dimensional pattern (namely a triangle) is needed in general to describe all the pairwise relations between three communities. The algorithmic details of choosing the number of embedding dimensions for LE, ISO and TREXPIC are provided in Sect. S3 of the Supplementary Information.

### Extraction of communities with weight thresholding

As described in Results, for demonstration purposes we implemented a really simple community detection method that performs a weight thresholding on the weighted networks obtained from the IERW process. Namely, we aimed at splitting a network into groups of densely connected nodes through the following steps:

1. Sort the weights of the *E* number of links of the embedded network in increasing order.
2. Remove the $\lfloor 0.05\cdot E\rfloor$ lowest and the largest links from the ordered list to ensure the removal of at least $5\%$ but at most $95\%$ of the links during the weight thresholding.
3. Find the largest gap between the consecutive link weights in the ordered list and set the weight threshold to the average of the two weight values on the sides of the largest gap.
4. Prune the examined graph.
  - When dealing with distance-like link weights that are smaller for stronger connections (i.e., when using LE, TREXPIC or ISO), remove the links having weights larger than the threshold.
  - When dealing with proximity-like link weights that are larger for stronger connections (i.e., when using node2vec), remove the links having weights smaller than the threshold.
5. Identify each of the connected components in the pruned graph as a community.

### Generating synthetic networks with communities using the planted partition model

The planted partition (PP) model^38^ is a special case of the stochastic block model (SBM)^52^, where there are only two values for the link probability: $p_{\textrm{in}}$, for pairs of nodes in the same community/block and $p_{\textrm{out}}$ for pairs of nodes in different communities/blocks. To generate networks with the PP model, we used the Python function ‘planted_partition_graph’ available in the ‘NetworkX’ package.

The input parameters of the model are the total number *N* of nodes, the number *m* of nodes in each community and the expected average degree ${\bar{\kappa }}$. In the above-presented measurements, following the settings in Ref.^26^, we used $N=1000$, $m=50$ (yielding $C=N/m=20$ communities) and ${\bar{\kappa }}=20$. To obtain community structures of different strengths, we tuned the mixing parameter ${\mu \in [0,1]}$, which we defined as the fraction between the expected number of neighbors of a randomly chosen node outside of its community and the expected total number of neighbors, i.e. as $\mu ={\bar{\kappa }}_{\textrm{out}}/{\bar{\kappa }}$. Given the mixing parameter $\mu$ and the expected average degree ${\bar{\kappa }}$, we calculated the expected number of inter-cluster edges of each node as ${\bar{\kappa }}_{\textrm{out}}=\mu \cdot {\bar{\kappa }}$, and the expected number of intra-cluster edges of each node as ${\bar{\kappa }}_{\textrm{in}}={\bar{\kappa }}-{\bar{\kappa }}_{\textrm{out}}$. Then, we derived the desired connection probabilities $p_{\textrm{out}}$ and $p_{\textrm{in}}$ from the formulas ${{\bar{\kappa }}_{\textrm{out}}=p_{\textrm{out}}\cdot (C-1)\cdot m}$ and ${\bar{\kappa }}_{\textrm{in}}=p_{\textrm{in}}\cdot (m-1)$. Self-loops are not included in the applied implementation, meaning that the number of possible neighbors of a node within its own block is $m-1$ instead of *m*. In our measurements, we used the settings $\mu =0.1,\,0.15,\,0.2,\,\ldots ,\,0.85,\,0.9$, where smaller values correspond to more easily detectable community structures.

To provide an example for a more complicated case, in Sect. S6 of the Supplementary Information we also utilise the ability of the stochastic block model to generate hierarchical community structures and show that IERW can facilitate the detection of the planted communities on both levels of the examined hierarchy.

### Generating synthetic networks with communities using the Lancichinetti–Fortunato–Radicchi benchmark

The Lancichinetti–Fortunato–Radicchi (LFR) benchmark^39^ generates graphs with power-law distributions of the node degrees and the community sizes, enabling the emergence of heterogeneity in these two quantities. The input parameters of the model are the total number *N* of nodes, the expected average degree ${\bar{\kappa }}$, the allowed largest degree $\kappa _{\textrm{max}}$, the exponent $\gamma$ of the tail of the degree distribution ($\mathcal {P}(\kappa )\sim \kappa ^{-\gamma }$), the allowed smallest and largest community sizes $m_{\textrm{min}}$ and $m_{\textrm{max}}$, the exponent $\beta$ of the tail of the community size distribution ($\mathcal {P}(m)\sim m^{-\beta }$), and the mixing parameter ${\mu \in [0,1]}$, having the same definition that we used in the case of the PP model, meaning that each node is expected to share a fraction of $1-\mu$ of its links with the other nodes of its own community and the remaining fraction $\mu$ with the nodes of the other communities. We examined LFR networks with non-overlapping clusters that we generated with the C++ code downloaded from https://www.santofortunato.net/resources. In the above measurements, following the settings in Ref.^26^, we used $N=1000$, ${\bar{\kappa }}=20$, $\kappa _{\textrm{max}}=50$, $\gamma =2.0$, $m_{\textrm{min}}=10$, $m_{\textrm{max}}=100$ and $\beta =3.0$, tuning the mixing parameter between $\mu =0.1$ (yielding easily detectable community structures with links falling mostly within communities) and $\mu =0.9$ (where most of the links connect nodes of different communities).

### Supplementary Information

Supplementary Information.

## Data Availability

All data generated during the current study are available from the corresponding author upon request.

## Code availability

The code used for performing the iterative embedding is available at https://github.com/BianKov/iterEmb.
