# Supplementary material for: Iterative embedding and reweighting of complex networks reveals community structure
Source: Sci Rep. 2024 Jul 26;14:17184. doi: 10.1038/s41598-024-68152-w (PMC11282304; doi:10.1038/s41598-024-68152-w)
Supplement: Supplementary file 1 — Supplementary Information. [file 41598_2024_68152_MOESM1_ESM.pdf]

Iterative embedding and reweighting of complex networks reveals community structure – Supplementary Information

Bianka Kovács[1], Sadamori Kojaku[2,3], Gergely Palla[4,1,*], and Santo Fortunato[2]

[1]Department of Biological Physics, Eötvös Loránd University, Pázmány P. stny. 1/A, Budapest, H-1117, Hungary
[2]Luddy School of Informatics, Computing, and Engineering, Indiana University, 1015 East 11th Street, Bloomington, 47408, Indiana, USA
[3]Department of Systems Science and Industrial Engineering, SUNY Binghamton, P.O. Box 6000, Binghamton, 13902, New York, USA
[4]Health Services Management Training Centre, Semmelweis University, Kútvölgyi út 2., Budapest, H-1125, Hungary
[*]gergely.palla@emk.semmelweis.hu

S1 The applied embedding algorithms in detail

This section provides the exact definition of the four node embedding algorithms that we examined. Section S1.1 describes Laplacian Eigenmaps (LE), Sect. S1.2 deals with the hyperbolic method named TRansformation of EXponential shortest Path lengths to hyperbolIC measures (TREXPIC), Sect. S1.3 presents Isomap (ISO) and its exponentialized version, and Sect. S1.4 details node2vec. At the end of each subsection, the weight formula applied with the given embedding algorithm in IERW is also explained. Note that while LE, TREXPIC and ISO expect distance-like link weights (where higher values refer to weaker connection), node2vec expects proximity-like link weights (where a higher value indicates the higher strength or relevance of the given connection).

S1.1 Node embedding with Laplacian Eigenmaps

The Laplacian Eigenmaps (LE) method was originally developed in Ref.[1] for mapping data points given in a high-dimensional space to a lower-dimensional space based on the eigendecomposition of the Laplacian matrix of a nearest neighbor graph created from the original data set. Considering a real complex network as a graph constructed in a high-dimensional space following the distance relations between the data points, the steps carried out in the LE algorithm after creating the neighborhood graph can be applied for obtaining a spatial representation of the network topology[2–6], yielding the following node embedding algorithm:

1. If link weights $w_{ij} \geq 0$ are given, these are interpreted by the algorithm as the distance between the connected nodes i, j in the latent high-dimensional space, meaning that a larger link weight is read as a weaker connection between two nodes. The inputted distance-like weights are converted to proximity-like weights $w'_{ij} \in (0,1]$ using the exponential formula

$$w'_{ij}(w_{ij}) = e^{-\frac{w_{ij}^2}{t}}, \tag{S1}$$

where, following the implementation created for Ref.[4], we calculated the scaling factor t as the square of the mean of the distance-like weights. When using our IERW process with the LE method, we re-set the t parameter in each iteration according to the current link weights.

2. The adjacency matrix $\boldsymbol{A}$ is created.

   (a) For unweighted graphs, $A_{ij} \equiv A_{ji} = 0$ if node i is not connected to node j and otherwise $A_{ij} \equiv A_{ji} = 1$.

   (b) For weighted graphs, $A_{ij} \equiv A_{ji} = 0$ if and only if nodes i and j are not connected to each other, and $A_{ij} \equiv A_{ji} = w'_{ij}$ otherwise. Note that links with 0 distance-like weight yield non-zero elements in the adjacency matrix $\boldsymbol{A}$ since $w'_{ij}(0) = 1$.

   As self-loops are not considered, $A_{ii} = 0$ for any node index i in both cases.

3. The Laplacian matrix $\boldsymbol{L} = \boldsymbol{\mathcal{D}} - \boldsymbol{A}$ is calculated, where $\boldsymbol{\mathcal{D}}$ is the diagonal matrix of elements $\mathcal{D}_{ii} = \sum_j A_{ij}$.

4. Given the generalized eigenvector problem $\boldsymbol{L} \cdot \underline{f}_\ell = \lambda_\ell \cdot \boldsymbol{\mathcal{D}} \cdot \underline{f}_\ell$, the eigenvectors $\underline{f}_1, \underline{f}_2, ..., \underline{f}_d$ corresponding to the eigenvalues $\lambda_1 \leq \lambda_2 \leq ... \leq \lambda_d$ are computed. The first eigenvalue λ_0 in the increasing order of eigenvalues is always 0, and the corresponding eigenvector $\underline{f}_0$ is not used in the embedding.

5. Each eigenvector is used as a list of Cartesian coordinates of all the embedded network nodes along a given axis of the d-dimensional Euclidean space: the ℓth ($\ell = 1, 2, ..., d$) Cartesian coordinate in the position vector $\underline{y}_i$ of the graph's i^{th} ($i = 1, 2, ..., N$) node is set to the ith element of the eigenvector $\underline{f}_\ell$, i.e. $\underline{y}_i(\ell) = \underline{f}_\ell(i)$.

For a network of N number of nodes, the computational complexity of embedding a network in the d-dimensional Euclidean space using the above algorithm is $\mathcal{O}\left((d+1) \cdot N^2\right)$, where the dominant step is the eigendecomposition of the graph Laplacian.

LE focuses on preserving local information, namely the neighborhood relations by putting the (strongly) connected nodes as close to each other in the embedding space as possible: it assigns to every node i a position vector $\underline{y}_i$ in such a way that a weighted sum $\sum_{i=1}^{N} \sum_{j=1}^{N} A_{ij} \cdot ||\underline{y}_i - \underline{y}_j||^2$ of the squared Euclidean distances between the connected node pairs is minimized while preventing the collapse of all the network nodes into a single point[1,5]. In our iterative embedding process, we made LE placing nodes with small angular distance $\Delta\theta_{ij}$ closer to each other with respect to the Euclidean distance in the subsequent iteration by using the formula

$$w_{ij} = 1 - \cos(\Delta\theta_{ij}), \tag{S2}$$

for creating distance-like link weights. Since placing nodes close to each other in the Euclidean space inherently makes their angular separation small, the iteration of LE using the weight formula of Eq. (S2) can increase the separation between the angularly arranged communities.

S1.2 Node embedding with TRansformation of EXponential shortest Path lengths to hyperbolIC measures

The method named TRansformation of EXponential shortest Path lengths to hyperbolIC measures (TREXPIC) was introduced in Ref.[7] for embedding networks in the hyperbolic space, even considering the possible directedness of the links. The hyperbolic space is commonly claimed to be a particularly good candidate for hosting graphs that have an underlying hierarchical orderliness or tree-like structure. The presence of hierarchy is usually understood broadly, as a general consequence of the presence of some kind of heterogeneity of the network nodes, which implies the possibility for classifying the nodes into groups that are interconnected through a containment hierarchy[8], or yields a natural ranking of the network nodes where the status of each node is determined by the given heterogeneous topological property[9] (e.g. the node degree, which can be treated as a simple measure of importance, and thus, as a proxy for a node's status in the hierarchy of importance[10]).

TREXPIC places the network nodes in the so-called native representation[8] of the hyperbolic space that visualizes the negatively curved hyperbolic space in the flat Euclidean space simply as a ball of infinite radius that we call the native ball or, in the two-dimensional case, the native disk. The aim of TREXPIC is the reconstruction of a matrix $\boldsymbol{X}$ describing node-node distances measured along the links of a network in the form of the matrix of pairwise hyperbolic distances between the embedded network nodes. The hyperbolic distance $x_{\underline{y},\underline{z}}$ between two points given by the Cartesian coordinate vectors $\underline{y} = \left[\underline{y}(1), \underline{y}(2), ..., \underline{y}(d)\right]$ and $\underline{z} = [\underline{z}(1), \underline{z}(2), ..., \underline{z}(d)]$ in the d-dimensional native ball can be calculated from the hyperbolic law of cosines written as

$$\cosh\left(\zeta x_{\underline{y},\underline{z}}\right) = \cosh(\zeta r_y)\cosh(\zeta r_z) - \sinh(\zeta r_y)\sinh(\zeta r_z)\cos(\Delta\theta_{y,z}), \tag{S3}$$

where $\zeta \in \mathbb{R}^+$ is connected to the curvature $K < 0$ of the hyperbolic space as $\zeta = \sqrt{-K}$, $\Delta\theta_{y,z} = \text{acos}\left(\frac{\underline{y} \cdot \underline{z}}{||\underline{y}|| \, ||\underline{z}||}\right) = \text{acos}\left(\frac{\sum_{\ell=1}^{d} \underline{y}(\ell)\underline{z}(\ell)}{r_y r_z}\right)$ is the angular distance between the given two points, while $r_y \equiv ||\underline{y}|| = \sqrt{\sum_{\ell=1}^{d} \underline{y}(\ell)^2}$ and $r_z \equiv ||\underline{z}|| = \sqrt{\sum_{\ell=1}^{d} \underline{z}(\ell)^2}$ denote the radial coordinate of the examined two points. According to Eq. (S3), $r_y = 0$ yields $x_{\underline{y},\underline{z}} = r_z$, and for $r_z = 0$ simply $x_{\underline{y},\underline{z}} = r_y$, meaning that in the native representation, the hyperbolic distance measured from the origin $\underline{o} = [0, 0, ..., 0]$ (which is the center of the native ball) is equal to the corresponding Euclidean distance, i.e. radial coordinate.

In addition to the native representation, TREXPIC also builds on the hyperboloid representation of the hyperbolic space in an intermediate step, just like a previous hyperbolic embedding method called hydra (hyperbolic distance recovery and approximation)[11]. The hyperboloid model represents the d-dimensional hyperbolic space in the $(d+1)$-dimensional Euclidean space as the upper sheet of a two-sheet hyperboloid. Here, the hyperbolic distance $x_{\underline{y},\underline{z}}$ between two points given by the Cartesian coordinate vectors $\underline{y} = \left[\underline{y}(1), \underline{y}(2), ..., \underline{y}(d+1)\right]$ and $\underline{z} = \left[\underline{z}(1), \underline{z}(2), ..., \underline{z}(d+1)\right]$ can be calculated as

$$x_{\underline{y},\underline{z}} = \text{acosh}\left(\underline{y} \circ \underline{z}\right)/\zeta, \tag{S4}$$

where $\underline{y} \circ \underline{z}$ is the Lorentz product

$$\underline{y} \circ \underline{z} = \underline{y}(1)\underline{z}(1) - \big(\underline{y}(2)\underline{z}(2) + \underline{y}(3)\underline{z}(3) + ... + \underline{y}(d+1)\underline{z}(d+1)\big) \tag{S5}$$

between the two position vectors. The first coordinate $\underline{y}(1)$ of a $(d+1)$-dimensional position vector $\underline{y}$ given in the hyperboloid representation is measured along the hyperboloid's axis of rotation, and is always positive in the case of the upper sheet. It can be connected based on the formula $x_{\underline{y},\underline{y}} \equiv 0$ — that is equivalent to $\underline{y} \circ \underline{y} \equiv 1$, as it can be seen from Eq. (S4) — to the length of the d-dimensional vector formed by the coordinates from the second to the $(d+1)$th one as $\sqrt{\sum_{\ell=2}^{d+1} \underline{y}(\ell)^2} = \sqrt{\underline{y}(1)^2 - 1}$. Besides, the first coordinate $\underline{y}(1)$ can be used for computing the hyperbolic distance between any position given by $\underline{y} \in \mathbb{R}^{d+1}$ and the origin of the hyperbolic space that is given in the hyperboloid representation by the position vector $\underline{o} = [1, 0, 0, ..., 0]$: according to Eqs. (S4) and (S5), $x_{\underline{y},\underline{o}} = \operatorname{acosh}\big(\underline{y} \circ \underline{o}\big)/\zeta = \operatorname{acosh}\big(\underline{y}(1) \cdot 1 - (\underline{y}(2) \cdot 0 + \underline{y}(3) \cdot 0 + ... + \underline{y}(d+1) \cdot 0)\big)/\zeta = \operatorname{acosh}\big(\underline{y}(1)\big)/\zeta$.

The detailed steps of the algorithm of TREXPIC for embedding an undirected network in the d-dimensional hyperbolic space of curvature $K = -\zeta^2$ (where $\zeta \in \mathbb{R}^+$) are the following:

1. Creation of the matrix $\boldsymbol{X}$ of expected pairwise hyperbolic distances between the embedded network nodes according to the exponential formula

$$X_{ij} \equiv X_{ji} = e^{-\frac{t}{\mathrm{SPL}_{ij}}} \in [0, 1), \tag{S6}$$

where the fast-changing, exponential mapping is intended to enable capturing the topological relations of the examined network more precisely.

   (a) For unweighted graphs, SPL_{ij} is the possible smallest number of hops in which the jth network node can be reached from the ith network node along the links.

   (b) If link weights $w_{ij} \geq 0$ are given, these are interpreted as the expected hyperbolic distance between the connected nodes i, j, meaning that a larger link weight is read as a weaker connection between two nodes, and two nodes connected with a link of 0 weight — which must not be confused with an unconnected node pair — can access each other the easiest. In this case, SPL_{ij} is the possible smallest sum of link weights along the paths that connect the ith network node to the jth one.

In both cases, $\mathrm{SPL}_{ii} = 0$, yielding $X_{ii} = 0$ for any node index i. Following the default setting of the implementation of TREXPIC published for Ref.[7], we calculated the multiplying factor t in Eq. (S6) from the largest shortest path length $\mathrm{SPL}_{\max}$ of the given network as $t = \sqrt{\ln(1.0/0.9999) \cdot \ln(1.0/0.1)} \cdot \mathrm{SPL}_{\max}$, corresponding to the geometric mean of two extreme settings given by $t_{\mathrm{small}} = \ln(1.0/0.9999) \cdot \mathrm{SPL}_{\max}$ (yielding a largest expected hyperbolic distance of 0.9999) and $t_{\mathrm{large}} = \ln(1.0/0.1) \cdot \mathrm{SPL}_{\max}$ (yielding a largest expected hyperbolic distance of 0.1). When using our IERW process with the TREXPIC method, we re-set the t parameter in each iteration according to the current $\mathrm{SPL}_{\max}$ yielded by the current link weights.

2. Conversion of the matrix $\boldsymbol{X}$ of expected hyperbolic distances to the matrix $\boldsymbol{\mathcal{L}}$ of the corresponding expected Lorentz products in the hyperboloid representation of the d-dimensional hyperbolic space of curvature $K = -\zeta^2$ according to Eq. (S4): $\mathcal{L}_{ij} = \cosh(\zeta \cdot X_{ij})$. Following the default setting in Ref.[7], we always set ζ to 1.

3. The derivation of the length and the direction of the position vector of each network node i ($i = 1, 2, ..., N$) in the d-dimensional native ball in accordance with the expected Lorentz products (and hereby also following the expected hyperbolic distances):

   i. Find the largest $d+1$ number of singular values $\sigma_1 \geq \sigma_2 \geq ... \geq \sigma_{d+1}$ and the corresponding singular vectors $\underline{u}_1, \underline{u}_2, ..., \underline{u}_{d+1}$ of the Lorentz product matrix $\boldsymbol{\mathcal{L}}$.

   ii. Use the largest singular value σ_1 and the corresponding singular vector $\underline{u}_1$ to calculate the ith node's hyperbolic distance from the origin of the hyperbolic space: $x_{i,\underline{o}} = \operatorname{acosh}\left(\sqrt{\sigma_1} \cdot \underline{u}_1(i)\right)/\zeta$. Remark that the singular values are always non-negative, and the singular vector $\underline{u}_1$ corresponds to the leading eigenvector of the matrix $\boldsymbol{\mathcal{L}} \cdot \boldsymbol{\mathcal{L}}^{\mathrm{T}}$, which eigenvector — according to the Perron–Frobenius theorem — can be chosen to have only positive components. If some numerical errors yield $\sqrt{\sigma_1} \cdot \underline{u}_1(i) < 1$, simply set $x_{i,\underline{o}}$ to $\operatorname{acosh}(1)/\zeta = 0$.

   iii. Compute a d-dimensional direction vector for the ith network node using the singular values and vectors from the second to the $(d+1)$th one: $\underline{e}_i = \left[\sqrt{\sigma_2} \cdot \underline{u}_2(i), \sqrt{\sigma_3} \cdot \underline{u}_3(i), ..., \sqrt{\sigma_{d+1}} \cdot \underline{u}_{d+1}(i)\right]/n$, where n is the norm $n = \sqrt{\left(\sqrt{\sigma_2} \cdot \underline{u}_2(i)\right)^2 + \left(\sqrt{\sigma_3} \cdot \underline{u}_3(i)\right)^2 + ... + \left(\sqrt{\sigma_{d+1}} \cdot \underline{u}_{d+1}(i)\right)^2}$.

Note that when embedding undirected networks, the Lorentz product matrix $\boldsymbol{\mathcal{L}}$ is symmetric, and thus, $\boldsymbol{U} = \boldsymbol{V}$ in the singular value decomposition $\boldsymbol{\mathcal{L}} = \boldsymbol{U} \cdot \boldsymbol{\Sigma} \cdot \boldsymbol{V}^{\mathrm{T}}$, where $\boldsymbol{\Sigma}$ is the diagonal matrix of singular values, and the columns of the matrixes $\boldsymbol{U}$ and $\boldsymbol{V}$ are the left and the right singular vectors of $\boldsymbol{\mathcal{L}}$, respectively. Besides, in the hyperboloid representation of the $(N-1)$-dimensional hyperbolic space, the $N \times N$-sized matrix $\boldsymbol{\mathcal{L}}$ of expected Lorentz products can be fully reconstructed according to its definition given by Eq. (S5) — i.e., as $\boldsymbol{\mathcal{L}} = \boldsymbol{\mathcal{Y}} \cdot \boldsymbol{J} \cdot \boldsymbol{\mathcal{Y}}^{\mathrm{T}}$ with $\boldsymbol{J} = \mathrm{diag}(+1, -1, -1, ..., -1)$ of size $N \times N$ — if the $N \times N$-sized node coordinate matrix $\boldsymbol{\mathcal{Y}}$ is defined based on the singular value decomposition $\boldsymbol{\mathcal{L}} = \boldsymbol{U} \cdot \boldsymbol{\Sigma} \cdot \boldsymbol{U}^{\mathrm{T}}$ as $\boldsymbol{\mathcal{Y}} = [\sqrt{\sigma_1} \cdot \underline{u}_1 \,, \mathrm{i} \cdot \sqrt{\sigma_2} \cdot \underline{u}_2 \,, \mathrm{i} \cdot \sqrt{\sigma_3} \cdot \underline{u}_3 \,, ..., \mathrm{i} \cdot \sqrt{\sigma_N} \cdot \underline{u}_N]$ with $\mathrm{i} = \sqrt{-1}$ denoting the imaginary unit. The imaginary multiplying factors in the above formula of $\boldsymbol{\mathcal{Y}}$ do not raise any issues since the direction described by the coordinates from the second one is the same when all of these coordinates are purely imaginary as if they all were real numbers. When embedding in the native ball of $d < N-1$ number of dimensions, the directions that have smaller contribution in the Lorentz products — i.e., where the singular value is smaller — are neglected, while the hyperbolic distance from the origin of the hyperbolic space is preserved in any number of dimensions by setting it always according to the largest singular value σ_1 and the corresponding singular vector $\underline{u}_1$. A similar approach is used by the hydra embedding algorithm[11] that also separates from each other a so-called "directional projection" and a "radial projection" of some $(d+1)$-dimensional vectors obtained from a decomposition of a matrix of expected Lorentz products.

4. Calculation of the ith ($i = 1, 2, ..., N$) network node's ℓ^{th} ($\ell = 1, 2, ..., d$) Cartesian coordinate in the native representation of the d-dimensional hyperbolic space as $\underline{y}_i(\ell) = x_{i,\underline{o}} \cdot \underline{e}_i(\ell)$.

The computational complexity of embedding a network of N number of nodes in the d-dimensional hyperbolic space with the above algorithm is dominated at $d+1 < \ln(N)$ by the calculation of all the shortest path lengths in the network, yielding $\mathcal{O}\left(\ln(N) \cdot N^2\right)$, while the computational complexity of the truncated SVD is $\mathcal{O}\left((d+1) \cdot N^2\right)$.

When combining our iterative embedding process with TREXPIC, just like in the case of LE, we defined the required distance-like link weights with the simple formula

$$w_{ij} = 1 - \cos(\Delta\theta_{ij}). \tag{S7}$$

This way, we made TREXPIC placing nodes that lie at small angular distance $\Delta\theta_{ij}$ from each other hyperbolically closer in the subsequent iteration. Nevertheless, as it is clearly shown by the common approximating formula[8]

$$x_{ij} \approx r_i + r_j + \frac{2}{\zeta} \cdot \ln\left(\frac{\Delta\theta_{ij}}{2}\right) \tag{S8}$$

derived from Eq. (S3), bringing nodes closer to each other hyperbolically goes hand in hand with decreasing their angular separation since the hyperbolic distance x_{ij} is a decreasing function of the angular distance $\Delta\theta_{ij}$. Thus, during the iteration of TREXPIC, the communities that are usually arranged along the angular coordinates in the native representation of the hyperbolic space become more and more separated.

S1.3 Node embedding with Isomap

The Isomap (ISO) method was originally proposed in Ref.[12] for finding a lower-dimensional representation of data points given in a higher-dimensional space based on the eigendecomposition of the shortest path length (SPL) matrix of a neighborhood graph constructed from the data set given in the high-dimensional input space. Similarly to the case of Laplacian Eigenmaps (LE), the application of ISO for embedding real-world networks relies on the principle that the given graph can be considered as a neighborhood graph created from some high-dimensional data. Nevertheless, rather than trying to preserve local information and grasp specifically the direct connections between the nodes like LE, ISO aims at preserving the global network topology directly by searching for such a node arrangement in the d-dimensional Euclidean space in which the pairwise Euclidean distances between the nodes are as close to the topological distances — given by the SPLs — measured along the network as it is possible in a d-dimensional representation.

Throughout this study, instead of the original algorithm that works with eigendecomposition[12], we followed the implementation published for Ref.[4], which is built on singular value decomposition (SVD), just like the above-described TREXPIC method. Thus, when embedding a network in the d-dimensional Euclidean space with ISO, we assigned to each network node a position vector of d number of Cartesian coordinates through the following steps:

1. Creation of the matrix $\boldsymbol{D}$ of expected pairwise Euclidean distances between the network nodes in the embedding space: $D_{ij} \equiv D_{ji} = \mathrm{SPL}_{ij}$.

    (a) For unweighted graphs, SPL_{ij} is the possible smallest number of hops in which the jth network node can be reached from the ith network node along the links.

(b) If link weights $w_{ij} \geq 0$ are given, these are interpreted as the distance between the connected nodes i, j in the hidden high-dimensional space, meaning that a larger link weight is read as a weaker connection between two nodes, and two nodes connected with a link of 0 weight — which must not be confused with an unconnected node pair — can access each other the easiest. In this case, SPL_{ij} is the possible smallest sum of link weights along the paths that connect the ith network node to the jth one.

In both cases, $\mathrm{SPL}_{ii} = 0$ for any node index i.

2. Conversion of the matrix $\boldsymbol{D}$ of expected Euclidean distances to the matrix $\boldsymbol{I}$ of the corresponding expected Euclidean dot products. Choosing the origin as the position of the center of mass of the network nodes (which choice does not influence the distances between the nodes in the embedding), this is done by

   i. constructing the matrix $\boldsymbol{S}$ of squared expected distances from the elements $S_{ij} = D_{ij}^2$,

   ii. subtracting the corresponding averages from the rows and the columns of $\boldsymbol{S}$ to create a doubly centered version $\boldsymbol{S}_{\mathrm{dc}}$ where the mean of all rows and also all columns is 0,

   iii. and finally, using the formula $\boldsymbol{I} = -\boldsymbol{S}_{\mathrm{dc}}/2$.

3. Calculation of the $N \times d$-sized matrix $\boldsymbol{Y}$ of Cartesian node coordinates that closely reproduce the expected dot products (and hereby also the expected Euclidean distances), i.e. that fulfills $\boldsymbol{I} = \boldsymbol{Y} \cdot \boldsymbol{Y}^{\mathrm{T}}$ as much as it is viable using the given number of dimensions. As it was described in Ref.[13], when searching for d-dimensional position vectors, then an optimal solution for minimizing the L2-norm of $\boldsymbol{I} - \boldsymbol{Y} \cdot \boldsymbol{Y}^T$ is to

   i. find the largest d number of singular values $\sigma_1 \geq \sigma_2 \geq ... \geq \sigma_d$ and the corresponding singular vectors $\underline{u}_1, \underline{u}_2, ..., \underline{u}_d$ of the dot product matrix $\boldsymbol{I}$

   ii. and compute the ℓth ($\ell = 1, 2, ..., d$) Cartesian coordinate in the position vector of the ith ($i = 1, 2, ..., N$) network node, i.e. the element in the ith row and ℓ^{th} column of the coordinate matrix $\boldsymbol{Y}$ as $\underline{y}_i(\ell) = \sqrt{\sigma_\ell} \cdot \underline{u}_\ell(i)$.

   Note that in the N-dimensional Euclidean space, the $N \times N$-sized coordinate matrix calculated according to the above steps can be written based on the singular value decomposition (SVD) of the dot product matrix $\boldsymbol{I} = \boldsymbol{U} \cdot \boldsymbol{\Sigma} \cdot \boldsymbol{V}^{\mathrm{T}}$ (where $\boldsymbol{\Sigma}$ is the diagonal matrix of singular values, and the columns of the matrixes $\boldsymbol{U}$ and $\boldsymbol{V}$ are the left and the right singular vectors of $\boldsymbol{I}$, respectively) as $\boldsymbol{Y} = \boldsymbol{U} \cdot \sqrt{\boldsymbol{\Sigma}}$ or, since for symmetric $\boldsymbol{I}$ matrixes obtained from undirected networks $\boldsymbol{U} = \boldsymbol{V}$, as $\boldsymbol{Y} = \boldsymbol{V} \cdot \sqrt{\boldsymbol{\Sigma}}$, yielding $\boldsymbol{Y} \cdot \boldsymbol{Y}^{\mathrm{T}} = \boldsymbol{U} \cdot \sqrt{\boldsymbol{\Sigma}} \cdot \left(\boldsymbol{V} \cdot \sqrt{\boldsymbol{\Sigma}}\right)^{\mathrm{T}} = \boldsymbol{U} \cdot \sqrt{\boldsymbol{\Sigma}} \cdot \sqrt{\boldsymbol{\Sigma}} \cdot \boldsymbol{V}^{\mathrm{T}} \equiv \boldsymbol{I}$, meaning that at $d = N$ the $N \times N$-sized dot product matrix $\boldsymbol{I}$ is fully reproduced.

The computational complexity of embedding N network nodes in the d-dimensional Euclidean space with the above algorithm is dominated at $d < \ln(N)$ by the calculation of all the shortest path lengths in the network, yielding $\mathcal{O}\left(\ln(N) \cdot N^2\right)$, while the computational complexity of the truncated SVD is $\mathcal{O}\left(d \cdot N^2\right)$.

To enable capturing the topological relations of a network more precisely, we developed a new version of ISO that we call Isomap with exponentialized shortest path lengths. Here, following the idea of LE and TREXPIC (see Eqs. (S1) and (S6), respectively), we applied a relatively fast-changing, exponential function on the original topological measures, namely instead of using simply $D_{ij} \equiv D_{ji} = \mathrm{SPL}_{ij}$, we defined the expected Euclidean distances in step 1 of the above algorithm of ISO as

$$D_{ij} = e^{-\frac{t}{\mathrm{SPL}_{ij}}} \in [0, 1), \tag{S9}$$

just like we calculated the expected hyperbolic distances from the pairwise shortest path lengths between the nodes in Eq. (S6). For the parameter t, we used the default setting applied in TREXPIC, namely $t = \sqrt{\ln(1.0/0.9999) \cdot \ln(1.0/0.1) \cdot \mathrm{SPL}_{\max}}$, where $\mathrm{SPL}_{\max}$ denotes the largest shortest path length occurring in the examined network. When using our IERW process with the exponentialized ISO method, we re-set the t parameter in each iteration according to the current $\mathrm{SPL}_{\max}$ yielded by the current link weights.

Note that by exponentializing in Eq. (S9) not directly the link weights but the measured shortest path lengths instead, the effect of the exponentialization becomes visible in the angular arrangement of the embedded nodes of not only weighted but also unweighted networks. In unweighted networks, where all the link weights are the same (e.g. 1), the exponentialization of each link weight individually corresponds to simply multiplying all the SPLs in the network with the same constant factor, which results in a simple rescaling of all the pairwise expected inner products that leads to a multiplication of all the singular values of the matrix $\boldsymbol{I}$ with a constant factor but does not change the direction of the position vectors. Therefore, to utilize the exponentialization in unweighted networks too, we opted in Eq. (S9) for exponentializing not the individual link weights but the shortest path lengths.

The impact of the exponentialization of the SPLs in ISO is demonstrated by Sect. S2, showing that this modification greatly enhances the increase in the communities' angular separation ratio $\langle\Delta\theta\rangle_{\text{inter}}/\langle\Delta\theta\rangle_{\text{intra}}$ during the embedding iterations and our iterative embedding process with the exponentialized version of Isomap can be successful in contracting the communities even with the application of the really simple distance-like link weight formula

$$w_{ij} = 1 - \cos(\Delta\theta_{ij}) \tag{S10}$$

also used in the case of iterating LE and TREXPIC (see Eqs. (S2) and (S7), respectively).

S1.4 Node embedding with node2vec

The node2vec method[14] generates d-dimensional Euclidean embeddings based on the presumption that the network topology can be thoroughly explored via random walks. In this method, the local environment around a node is described through multiple truncated random walks along the links that yield ordered node lists having a given, restricted length. These node lists are used for embedding the nodes by following the same idea that was utilized in the word2vec algorithm[15] for creating spatial arrangements of words representing their similarities from the point of view of their typical contexts in given texts. node2vec replaces the "sentences" (or, more precisely, the context windows, i.e., the considered lists of consecutive words) with lists of consecutive nodes within random walks, trains an artificial neural network to learn the characteristic neighborhood of the network nodes and assigns spatial positions with small distances to those node pairs that frequently appear close to each other in the random paths. In our study, following Ref.[16], we always set the number of walks started from each node to 80, the length of each random walk to 10 and the length of the considered windows to 10.

The random walkers follow different node-node transition probabilities in node2vec, controlled by the return parameter p and the in-out parameter q, setting the non-normalized transition probabilities to

- $1/p$ for stepping back to that node from which the walker just arrived to the current node,
- 1 for stepping to a common neighbor of the current node and the previous one,
- and $1/q$ for the other cases, i.e. for moving away ("outward") from the preceding node.

Low values of p and high values of q bias the walks to be more local, proceeding less to farther nodes from a given starting node, resulting in embeddings that preserve well the explored neighborhood relations, while at high values of p and low values of q node2vec generates node arrangements focusing more on the global network topology. In our measurements, we always used the settings $p = 1$ and $q = 1$.

If link weights are given in the network to be embedded, node2vec modifies the transition probabilities accordingly: here a larger link weight is interpreted as a stronger connection, yielding a higher transition probability between the given two nodes. A simple choice for proximity-like link weights based on embeddings obtained from node2vec in our iterative embedding process is given by

$$w_{ij} = \cos(\Delta\theta_{ij}) + 1, \tag{S11}$$

where $\Delta\theta_{ij}$ denotes the angular distance between the ith and the jth network nodes and the addition of 1 ensures that all the link weights are non-negative (namely, $w_{ij} \in [0,2]$). However, following the idea of Laplacian Eigenmaps and TREXPIC about including an exponentialization step in the embedding process, to emphasize the differences between the simple link weights given by Eq. (S11), we applied a rather fast-changing function on these, and defined exponential link weights in the IERW process as

$$w_{ij} = e^{t\cdot\left(\cos(\Delta\theta_{ij})-1\right)}, \tag{S12}$$

where we replaced the original $+1$ term with a -1 in order to exponentialize non-positive values instead of non-negative ones and avoid numerical (overflow) errors this way. Note that the exponential link weights given by Eq. (S12) are still non-negative (fall in the range $[e^{-2t}, 1]$), and thus, can be interpreted as transition probabilities. We show the advantageous effect of using the exponential link weight formula in Eq. (S12) instead of Eq. (S11) in Sect. S2. Assuming that the heterogeneity of the degree distribution plays an important role from the viewpoint of the transition probabilities in random walks and that it can be well described by the ratio between the average node degree $\bar{\kappa}$ and the most frequent node degree, i.e. the mode $\hat{\kappa}$ of the degrees, we set the parameter t to $t = 10\cdot\bar{\kappa}/\hat{\kappa}$. If the mode of the degrees was not unique, we set $\hat{\kappa}$ to the smallest one of the most frequently occurring node degrees. Unlike in the case of LE (Eq. (S1)), TREXPIC (Eq. (S6)) and the exponentialized version of ISO (Eq. (S9)), where we re-set the t parameter in each iteration according to the current link weights, in the case of node2vec (Eq. (S12)) we used the same value of t at each iteration for a given network, namely the one that we chose based on the original, unweighted graph, considering only the number of connections and not node strengths.

The time complexity of node2vec for embedding a network of N nodes and E edges into a d-dimensional space is $\mathcal{O}\left(E+N\cdot d\cdot\omega^2\right)$, where ω denotes the window length, which was set to $\omega=10$ in our measurements. It is important to note that due to the application of random walks, node2vec is a stochastic embedding method, yielding different node arrangements when re-run for the same network. Nevertheless, since we repeated each of our measurements for multiple network samples anyway, we ran IERW with node2vec only once for each network.

S2 The beneficial effect of exponentialization in Isomap and node2vec

As it is shown by Eqs. (S1) and (S6) in Sect. S1, both Laplacian Eigenmaps[1] and TREXPIC[7] perform an exponentializing step when embedding a network. Expecting that such relatively fast-changing measures of the topological proximity and distance can effectively emphasize the differences between the relations of different node pairs and thereby improve the embedding performance, we wanted to include some exponentialization in the proposed Iterative Embedding and ReWeighting (IERW) process in the case of Isomap[12] and node2vec[14] too. Therefore, as it is detailed in Sects. S1.3 and S1.4, we created a modified version of Isomap by defining the expected Euclidean distances in Eq. (S9) as exponentialized shortest path lengths, and included the exponentialization in the iteration of node2vec by choosing for it in IERW an exponential link weight function given by Eq. (S12).

This section demonstrates the beneficial effect of introducing exponentialization in the IERW process in the case of Isomap and node2vec by comparing the angular separation ratios achieved with the exponentialized and the non-exponentialized versions on the networks examined in Figs. 3 and 4 of the main text. In the case of Isomap, the not exponential version corresponds to the original algorithm[12], where the expected Euclidean distances are defined as the simple shortest path lengths. In the case of IERW with node2vec, we defined the non-exponentialized version by replacing the exponential link weight function in the reweighting steps with the linear link weight function given by Eq. (S11).

Figure S1 supplements Fig. 3 of the main text, comparing the exponentialized and the non-exponentialized embedding iterations on planted partition networks[17]. Figure S2 supplements Fig. 4 of the main text, showing the same comparison for Lancichinetti–Fortunato–Radicchi networks[18]. The advantage of the exponentialized versions is striking for both types of test networks. Note that in the case of Isomap, at the smaller mixing parameters the first embedding already performs slightly better when using the exponentialization, which is due to our choice of exponentializing not the individual link weights (that are initially all equal to 1) but the shortest path lengths instead.

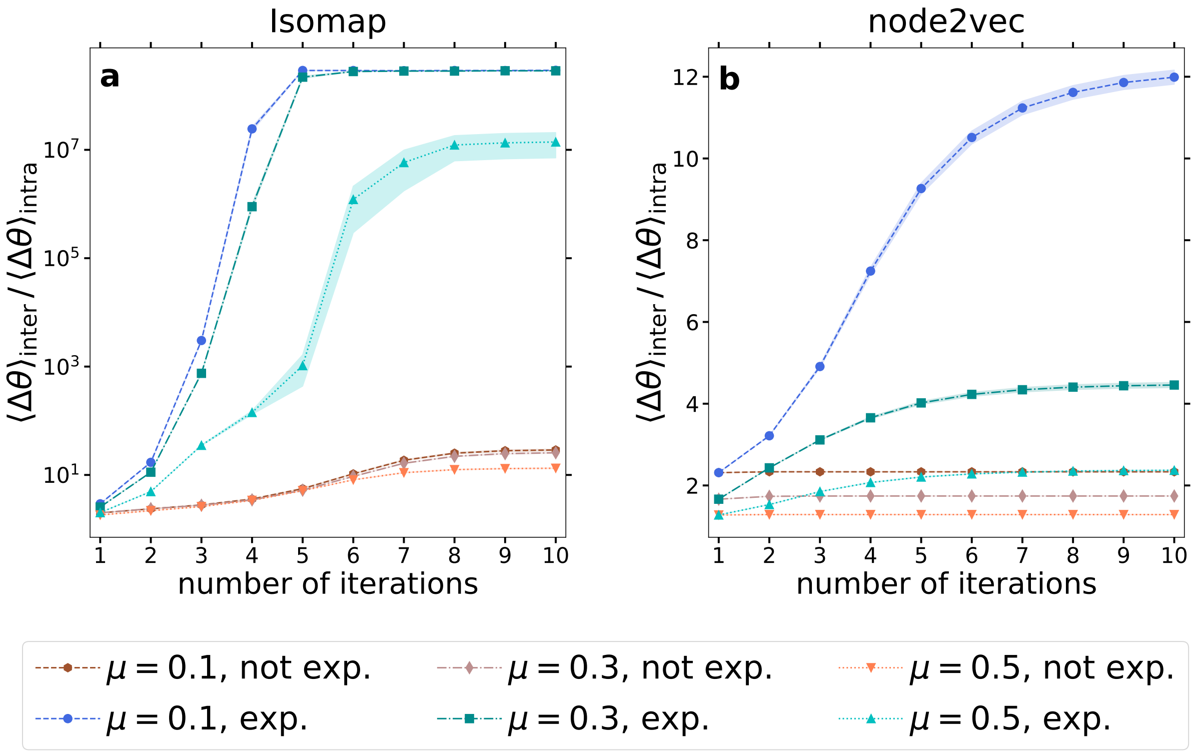

Figure S1. The beneficial effect of exponentialization in the iteration of Isomap (panel a) and node2vec (panel b) on the increase in the angular separation of the communities in planted partition networks. $\langle\Delta\theta\rangle_{\text{inter}}$ denotes the average of the angular distances over all possible node pairs of different communities, while $\langle\Delta\theta\rangle_{\text{intra}}$ stands for the average of the angular distances over all possible node pairs belonging to the same community. The curves of different line styles correspond to different values of the mixing parameter μ, yielding community structures of different detectability levels. The blue and green curves that refer to the exponentialized versions are the same as in panels **c** and **d** of Fig. 3 in the main text. The network generation parameters are given in the Methods section of the main text. Each depicted data point corresponds to the result averaged over 100 networks of the given parameter setting and the shaded areas show the standard error of the means.

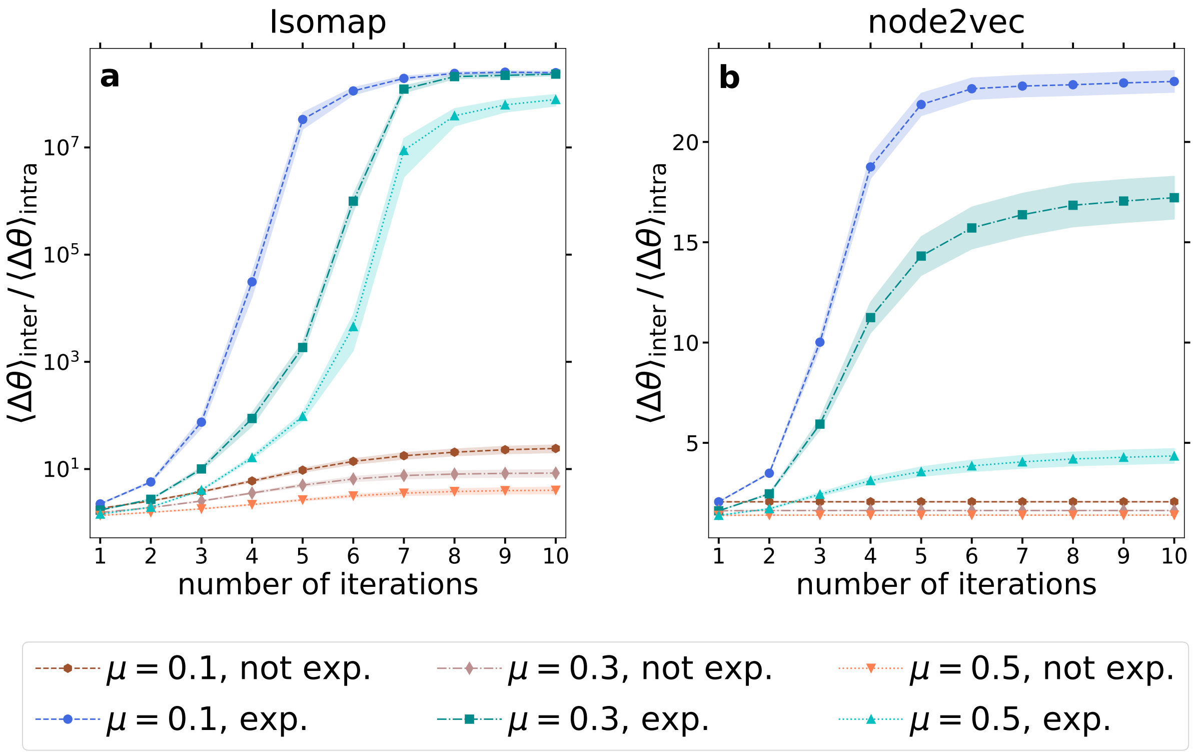

Figure S2. The beneficial effect of exponentialization in the iteration of Isomap (panel a) and node2vec (panel b) on the increase in the angular separation of the communities in Lancichinetti–Fortunato–Radicchi networks. $\langle\Delta\theta\rangle_{\text{inter}}$ denotes the average of the angular distances over all possible node pairs of different communities, while $\langle\Delta\theta\rangle_{\text{intra}}$ stands for the average of the angular distances over all possible node pairs belonging to the same community. The curves of different line styles correspond to different values of the mixing parameter μ, yielding community structures of different detectability levels. The blue and green curves that refer to the exponentialized versions are the same as in panels **c** and **d** of Fig. 4 in the main text. The network generation parameters are given in the Methods section of the main text. Each depicted data point corresponds to the result averaged over 100 networks of the given parameter setting and the shaded areas show the standard error of the means.

S3 Embedding parameters

This section deals with the settings of the parameters of the examined four embedding methods. First, Sect. S3.1 describes in detail how the number of embedding dimensions was set. Then, Sect. S3.2 considers the other parameter that can be tuned in all the cases, namely the scaling factor in the exponentializing step. Finally, Sect. S3.3 demonstrates that the applied parameter choices are (although typically not the optimal but) relatively favorable, falling in a reasonable range of the parameter space.

S3.1 Choosing the number of embedding dimensions

In the case of node2vec, according to our measurements shown in Sect. S3.3, the optimal number of embedding dimensions for revealing the community structure is not related strongly to the number of communities planted in a network, and the main requirement of node2vec is simply using a high enough number of dimensions. Therefore, to ensure a substantial reduction in the number of embedding dimensions d compared to the number of network nodes but also utilize the benefits of a relatively high-dimensional space, we always used node2vec with the setting $d = 64$, which is one of the standard choices in the literature.

Nevertheless, based on the measurements presented in Sect. S3.3, the matrix decomposition methods given by LE, TREXPIC and ISO are more sensitive to the number of dimensions of the embedding space. In general, it can be stated that node embeddings can grasp more information and thereby describe the local peculiarities of a network topology more precisely if the number of dimensions of the embedding space is higher. However, when aiming at the exploration of the communities of the network nodes, i.e. when focusing on the network structure at a mesoscopic scale instead of the individual pairwise interactions, using an excessive number of dimensions can be problematic too. When performing a dimension reduction of a matrix that characterizes the network topology, one has to separate from each other the important components and those that are rather redundant from the viewpoint of the given task. We assume that the number of variables necessary for properly describing the community structure is independent of the specific type of the matrix that is reduced in the embedding.

To find the proper number of variables from the viewpoint of community detection, let us consider the eigendecomposition $\boldsymbol{L}_{\text{norm}} \cdot \underline{g}_\ell = \lambda_\ell \cdot \underline{g}_\ell$ of the normalized Laplacian matrix

$$\boldsymbol{L}_{\text{norm}} = \boldsymbol{\mathcal{D}}^{-1/2} \cdot \boldsymbol{L} \cdot \boldsymbol{\mathcal{D}}^{-1/2} = \boldsymbol{\mathcal{D}}^{-1/2} \cdot (\boldsymbol{\mathcal{D}} - \boldsymbol{A}) \cdot \boldsymbol{\mathcal{D}}^{-1/2} \tag{S13}$$

of an undirected graph with an adjacency matrix $\boldsymbol{A}$ and a diagonal matrix $\boldsymbol{\mathcal{D}}$, with $\mathcal{D}_{ii} = \sum_j A_{ij}$. It is well known that here the smallest eigenvalue λ_{min} is always 0, and the most meaningful components are given by the eigenvectors belonging to the smallest non-zero eigenvalues. The question is how many eigenvectors should be retained, separating them from the less relevant eigenvectors that describe only local peculiarities. Considering simply the largest gap between consecutive elements in the increasing order of the (non-zero) eigenvalues as the point of transition from the truly meaningful "small" (but non-zero) eigenvalues to the less important "large" ones, the number of non-zero eigenvalues below this transition point can be a good proxy for the proper number of variables to be retained for revealing the community structure of a network. The idea that this selection between the eigenvalues may be suitable for grasping the main properties of the network topology in relation to the communities is supported by the fact — described e.g. in Ref.[19] — that while the multiplicity of the 0 eigenvalue is equal to the number of connected components in the graph (which is 1 in our measurements), the number of eigenvalues being either 0 or close to 0 is the same as the number of groups the network nodes can be partitioned into via sparse cuts, indicating that the number of small eigenvalues of the matrix $\boldsymbol{L}_{\text{norm}}$ and the number of communities in a network are strongly connected to each other.

Therefore, to set the proper number of embedding dimensions for LE, TREXPIC and ISO, we simply considered all the non-zero eigenvalues of a normalized graph Laplacian falling below the largest gap in the increasing order of the non-zero eigenvalues to be important. More specifically, we chose the number of embedding dimensions d of LE, TREXPIC and ISO through the following steps:

1. Pre-weight the graph to facilitate the selection of d.

   i. Assign a distance-like weight to each edge $i - j$ in the network following a repulsion-attraction rule (RA1) proposed in Ref.[4] for describing neighborhood topological information and the trade-off of the attraction between nodes having a high number of common neighbors and the repulsion between nodes having a lot of connections in distinct neighborhoods:

   $$w_{ij} = \frac{\kappa_i + \kappa_j + \kappa_i \cdot \kappa_j}{1 + \text{CN}_{ij}}, \tag{S14}$$

   where κ_i and κ_j stand for the total number of links connected to node i and node j, respectively, and CN_{ij} denotes the number of common neighbors of nodes i and j. Here a large link weight can emerge due to the presence of a relatively high number of neighbors not in common, indicating a more vulnerable connection or a larger topological distance.

ii. Convert the obtained distance-like link weights to proximity-like ones using the formula applied in the LE algorithm[1] (see Eq. (S1))

$$w'_{ij}(w_{ij}) = e^{-\frac{w_{ij}^2}{t}}, \tag{S15}$$

setting the scaling factor t to the square of the mean of the weights, as it was suggested in the LE implementation created for Ref.[4].

When working with a weighted graph, instead of the above-described artificial link weights, the actual weights can also be utilized. If all weights are non-negative and higher values mean stronger or more relevant connections, these can be used without modification. If, however, higher link weights correspond to larger topological distances, these can be converted to proximity-like link weights e.g. with the exponential formula given by Eq. (S15). Note that we do not use the pre-weights to do the embeddings, but only to set the number of dimensions of the embedding space.

2. Create the normalized Laplacian matrix $\boldsymbol{L}_{\text{norm}}$ of the pre-weighted graph according to Eq. (S13).

3. Compute the smallest $\lfloor N/2 \rfloor + 1$ number of eigenvalues of the normalized graph Laplacian $\boldsymbol{L}_{\text{norm}}$, where the choice $\lfloor N/2 \rfloor + 1$ sets the allowed largest number of embedding dimensions to $d_{\text{max}} = \lfloor N/2 \rfloor$ and thereby ensures a significant dimension reduction compared to the total number of network nodes N.

4. Sort the eigenvalues in increasing order, omitting the 0 eigenvalue to ensure that only the gaps between the non-zero eigenvalues will be taken into account.

5. Find the largest gap between consecutive eigenvalues in the obtained ordered list of $\lfloor N/2 \rfloor$ elements and perform a Modified Thompson Tau test with a significance level of $\alpha = 10^{-6}$ to decide whether the largest gap size can be considered to be an outlier among all the examined differences of successive eigenvalues.

   (a) If the largest gap size can be considered as an outlier, and thus, as a significant eigengap, set the number of embedding dimensions d to the number of elements of the examined list below the largest eigengap, yielding $d \in [1, \lfloor N/2 \rfloor - 1]$. If we get $d = 1$, we use $d = 2$.

   (b) Otherwise, consider the largest gap to be irrelevant and — assuming that there is no significant community structure in the given network — use $d = d_{\text{max}}$.

As it is demonstrated by Fig. S3, for not too large values of the mixing parameter μ, the number of dimensions d chosen by the above algorithm is a good indicator of the number of communities C planted in the investigated synthetic networks, namely usually the chosen number of embedding dimensions was $d = C - 1$. This fits the intuition since e.g. to describe all the pairwise relations between three communities, a two-dimensional pattern (i.e., a triangle) is needed in general.

S3.2 Choosing the scaling factor in the exponentializing step

As it is explained in Sect. S1, all four examined cases of IERW include an exponentializing step, either during the node embedding (when using LE, TREXPIC or the exponentialized version of ISO) or in the link weighting phase (in the case of IERW with node2vec). All the applied exponential formulas contain a tunable parameter given by the scaling factor t.

First, the algorithm of Laplacian Eigenmaps starts with an exponential conversion of the inputted distance-like link weights w_{ij} to proximity-like ones as $w'_{ij} = e^{-w_{ij}^2/t}$ (see Eq. (S1) in Sect. S1.1). Here, larger values of the parameter $t > 0$ blur the differences between the inputted distances more. At the extreme, $t = \infty$ treats all links equally, regardless of the corresponding distances. If not mentioned otherwise, we followed the implementation created for Ref.[4], and calculated the scaling factor t in LE as the square of the mean of the inputted distance-like weights.

Second, TREXPIC builds on an exponential distance, using $e^{-t/\text{SPL}_{ij}} \in [0,1)$ as the expected hyperbolic distance between nodes i and j (see Eq. (S6) in Sect. S1.2). Here, the multiplying factor $t > 0$ controls the speed of the increase in the expected hyperbolic distance with the increase in the shortest path length (SPL). For small enough values of the parameter t, $e^{-t/\text{SPL}_{ij}} \approx 1 - t/\text{SPL}_{ij}$ and all the non-zero SPLs are converted to expected hyperbolic distances close to 1, while the increase in t shifts the non-unit expected distances (corresponding to finite SPLs) towards 0. With the intention of avoiding any extreme settings, we followed Ref.[7] and calculated the default value of t from the occurring largest shortest path length SPL_{max} as $t = \sqrt{\ln(1.0/0.9999) \cdot \ln(1.0/0.1)} \cdot \text{SPL}_{\text{max}}$, corresponding to the geometric mean of two extreme settings given by $t_{\text{small}} = \ln(1.0/0.9999) \cdot \text{SPL}_{\text{max}}$ (yielding a largest expected hyperbolic distance of 0.9999) and $t_{\text{large}} = \ln(1.0/0.1) \cdot \text{SPL}_{\text{max}}$ (yielding a largest expected hyperbolic distance of 0.1).

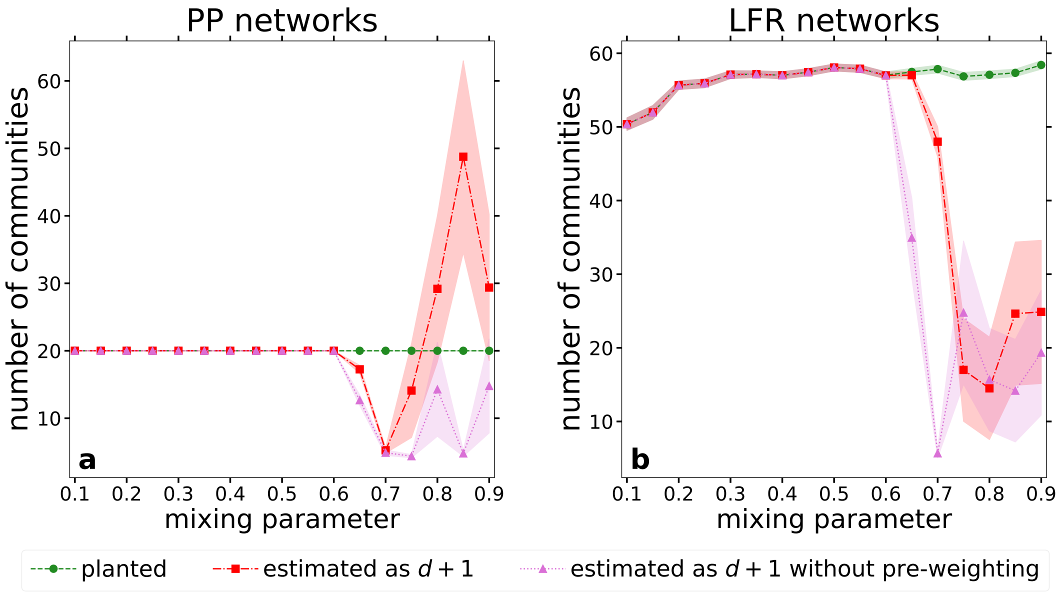

Figure S3. The relation between the number of embedding dimensions chosen by the above algorithm and the number of planted communities in synthetic networks. Panel **a** examines the planted partition graphs that were also studied e.g. in Fig. 3 of the main text, and panel **b** deals with the Lancichinetti–Fortunato–Radicchi networks that were also investigated in Fig. 4 of the main text. To confirm the beneficial effect of the pre-weighting step, the d values chosen based on the eigengaps of the graph Laplacian of the original, not weighted graphs are depicted too. Each data point corresponds to the average over 100 networks of the given parameter settings and the error bars show the standard error of the mean.

In the case of Isomap, we introduced an exponetialization mimicking TREXPIC, setting the expected Euclidean distance between nodes i and j to the exponential distance $e^{-t/\mathrm{SPL}_{ij}} \in [0,1)$ (see Eq. (S9) in Sect. S1.3). Since using exactly the same exponential formula, we transferred the default t value from TREXPIC to the exponentialized version of Isomap too.

Finally, when iterating node2vec, we utilized exponentialization not during the embedding but in the link weighting step and calculated the proximity-like link weights after each embedding from the angular distances $\Delta\theta_{ij} \in [0,\pi]$ as $w_{ij} = e^{t\cdot\left(\cos(\Delta\theta_{ij})-1\right)}$ (see Eq. (S12) in Sect. S1.4). Here, the multiplying factor $t>0$ controls the speed of the decrease in the random walk transition probability with the increase in the angular distance: at larger values of t, the transition probabilities decay faster as a function of $\Delta\theta_{ij}$. Assuming that the heterogeneity of the number of connections per node (i.e., the number of possible directions in which a random walk can be continued) plays an important role from the viewpoint of the transition probabilities in random walks, if not mentioned otherwise, we set the parameter t to $t = 10\cdot\bar{\kappa}/\hat{\kappa}$, where $\bar{\kappa}$ is the average node degree and $\hat{\kappa}$ is the most frequent node degree. If the mode of the degrees was not unique, we set $\hat{\kappa}$ to the smallest one of the most frequently occurring node degrees.

S3.3 Validating the choice of the embedding parameters

Figures S4-S7 demonstrate how the change in the number of embedding dimensions d and the scaling parameter t of the exponentializing step affects the angular separation achieved between communities in iterated embeddings. In all the figures, d^* denotes the number of embedding dimensions chosen according to the algorithm described in Sect. S3.1 (approximating $C-1$, where C is the number of communities planted in the given test network), and the "default" scaling factor corresponds to the setting described in Sect. S3.2. We used our usual stopping criterion at all the settings, meaning that the iteration was terminated when the relative change in the average link weight between subsequent iterations dropped below 0.001. Note that the number of iterations was limited to 20 in order to reduce the computational time. At the most extreme parameter settings, sometimes the matrix factorization has failed due to numerical errors — in these cases, we used the last successful embedding. After reaching the final embedding, we measured the ratio between the average inter-community angular distance $\langle\Delta\theta\rangle_{\mathrm{inter}}$ (i.e., the average of the angular distances over all the node pairs of different communities) and the average intra-community angular distance $\langle\Delta\theta\rangle_{\mathrm{intra}}$ (i.e., the average of the angular distances over all the node pairs belonging to the same community).

Considering the number of embedding dimensions, when comparing Figs. S4–S6 to Fig. S7, it is conspicuous that the performance of LE, TREXPIC and the exponentialized version of ISO shows multiple orders of difference as a function of d, while (in the vicinity of the default scaling factor) node2vec shows relatively weak d-dependence along the whole examined

range. According to Figs. S4–S6, our choice d^* always fell in the range of high performance (even when this range was very tight), justifying the application of our d-selecting algorithm detailed in Sect. S3.1. Regarding the scaling factor t in the exponentializing steps, our default settings seem to be adequate in general.

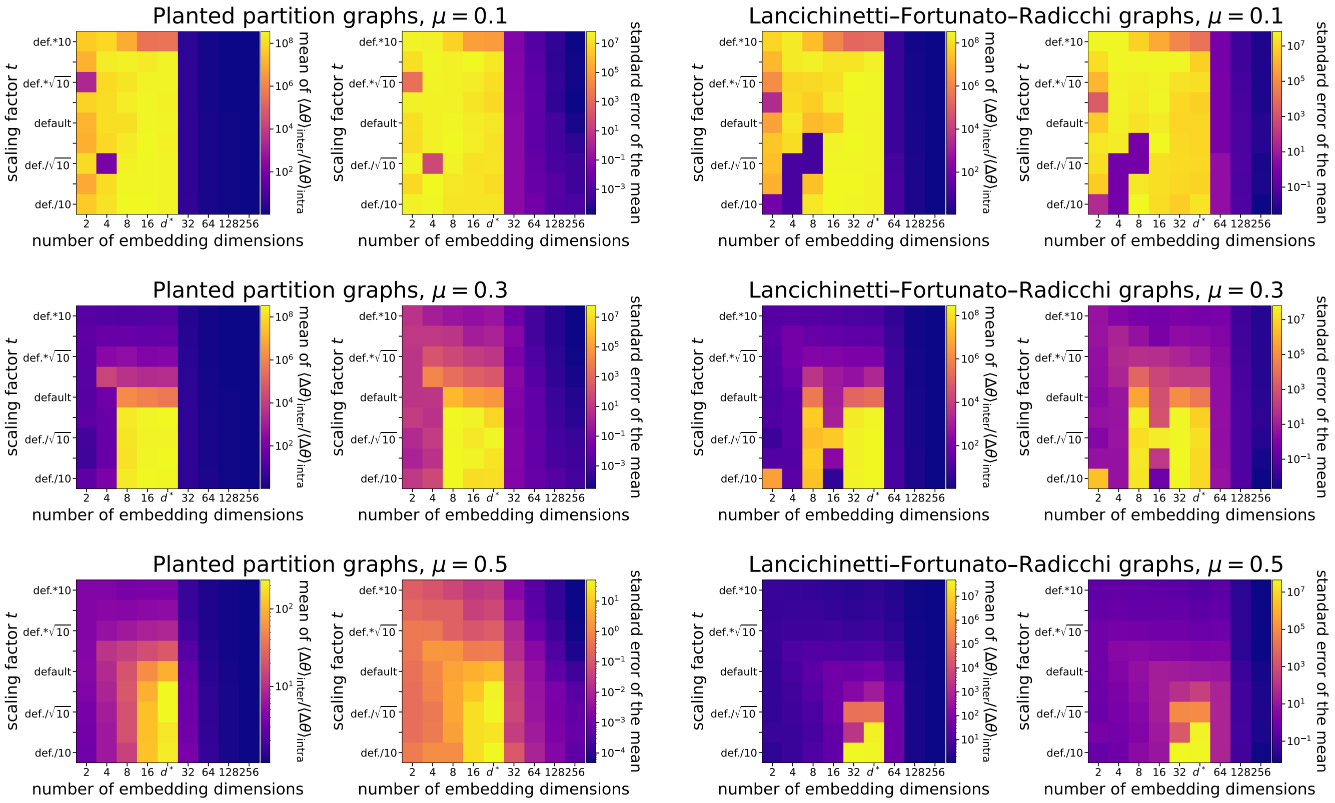

Figure S4. The effect of the embedding parameters on the ratio between the average inter-community angular distance $\langle\Delta\theta\rangle_{\text{inter}}$ and the average intra-community angular distance $\langle\Delta\theta\rangle_{\text{intra}}$ at the final step when iterating Laplacian Eigenmaps. Each pair of subplots depicts the results for 10 realizations of a given type of synthetic networks: the left half of the figure refers to planted partition graphs (also examined in Fig. 3 of the main text), while the panels on the right deal with Lancichinetti–Fortunato–Radicchi networks (also examined in Fig. 4 of the main text). The IERW procedure was performed only once for each network. The default scaling factor was set according to Ref.[4], and d^* is the number of embedding dimensions chosen by our algorithm described in Sect. S3.1.

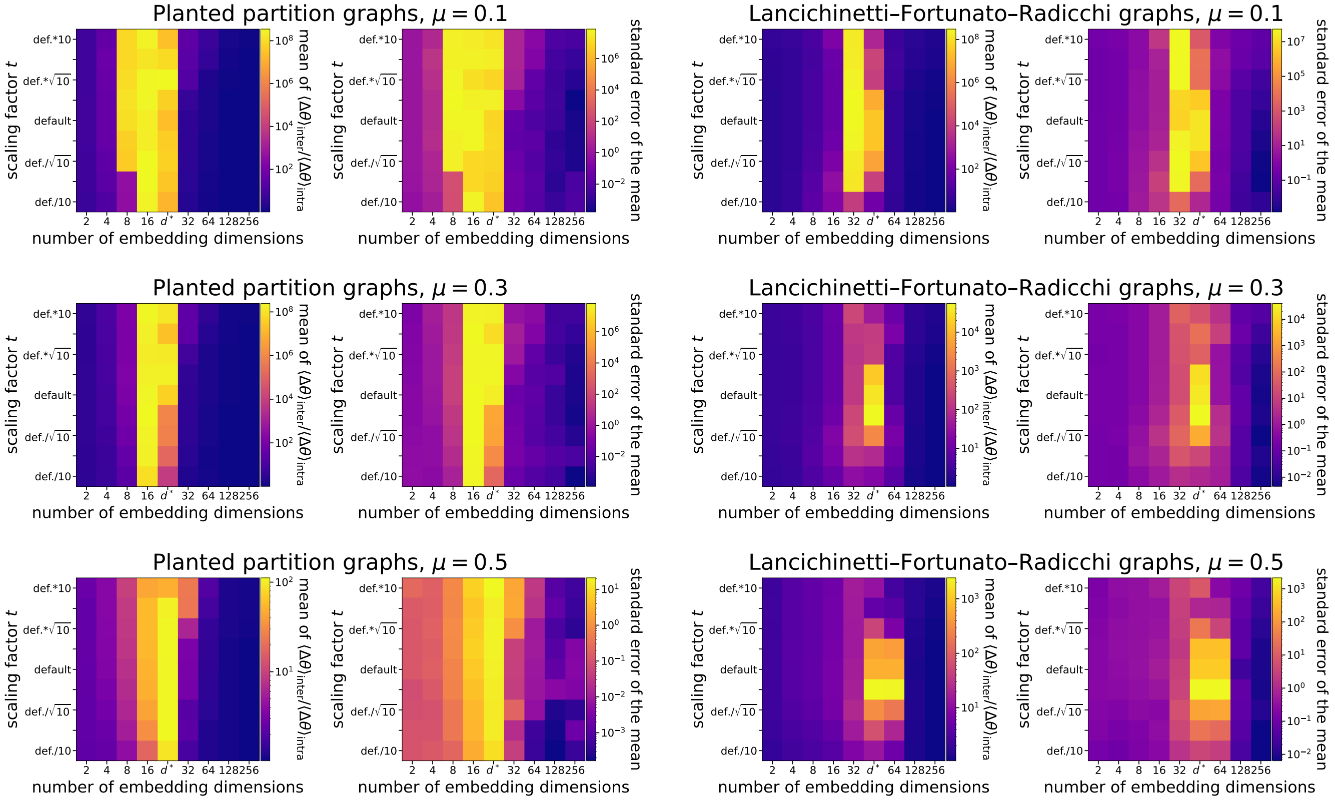

Figure S5. **The effect of the embedding parameters on the ratio between the average inter-community angular distance $\langle \Delta\theta \rangle_{\mathrm{inter}}$ and the average intra-community angular distance $\langle \Delta\theta \rangle_{\mathrm{intra}}$ at the final step when iterating TRansformation of EXponential shortest Path lengths to hyperbolIC measures.** Each pair of subplots depicts the results for 10 realizations of a given type of synthetic networks: the left half of the figure refers to planted partition graphs (also examined in Fig. 3 of the main text), while the panels on the right deal with Lancichinetti–Fortunato–Radicchi networks (also examined in Fig. 4 of the main text). The IERW procedure was performed only once for each network. The default scaling factor was set according to Ref.[7], and d^* is the number of embedding dimensions chosen by our algorithm described in Sect. S3.1.

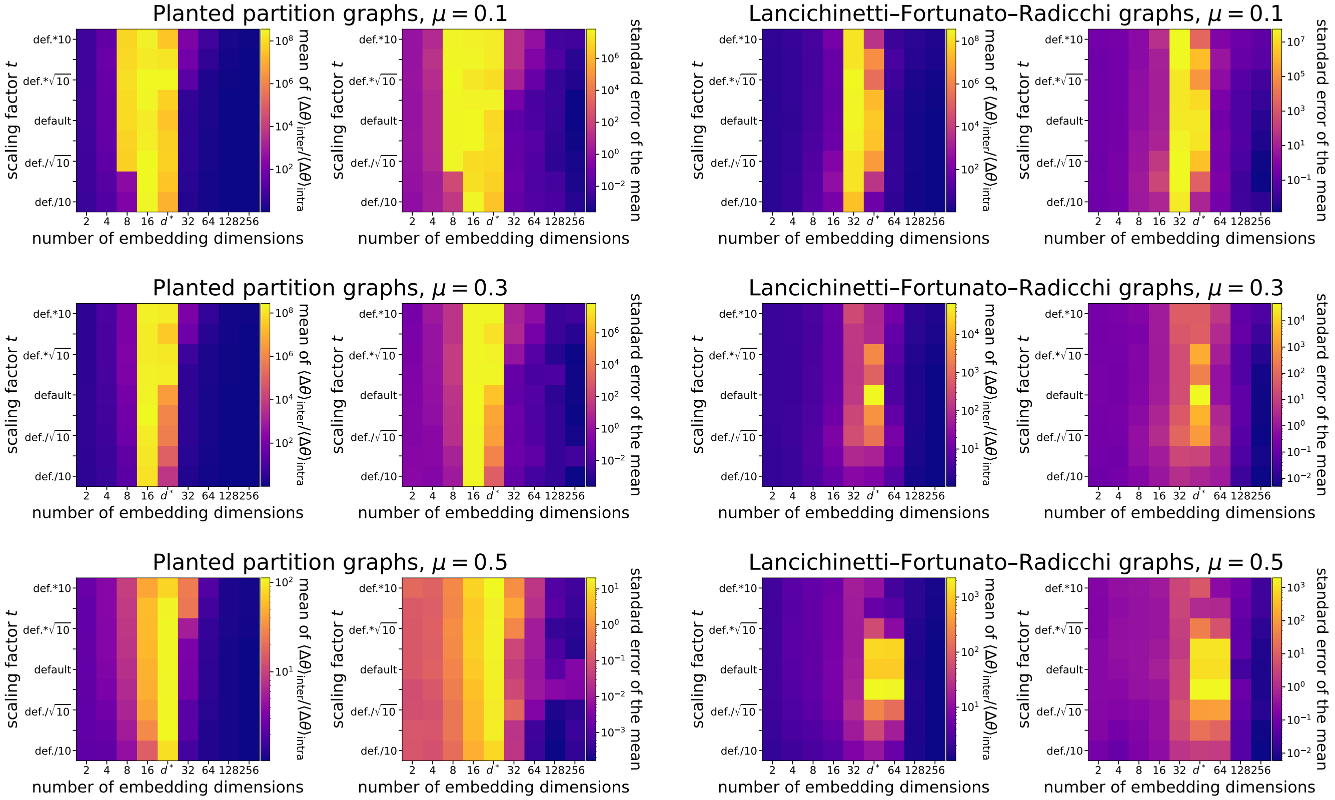

Figure S6. **The effect of the embedding parameters on the ratio between the average inter-community angular distance $\langle\Delta\theta\rangle_{\text{inter}}$ and the average intra-community angular distance $\langle\Delta\theta\rangle_{\text{intra}}$ at the final step when iterating Isomap with exponentialized shortest path lengths.** Each pair of subplots depicts the results for 10 realizations of a given type of synthetic networks: the left half of the figure refers to planted partition graphs (also examined in Fig. 3 of the main text), while the panels on the right deal with Lancichinetti–Fortunato–Radicchi networks (also examined in Fig. 4 of the main text). The IERW procedure was performed only once for each network. The default scaling factor was set according to our formula defined in Sect. S3.2, and d^* is the number of embedding dimensions chosen by our algorithm described in Sect. S3.1.

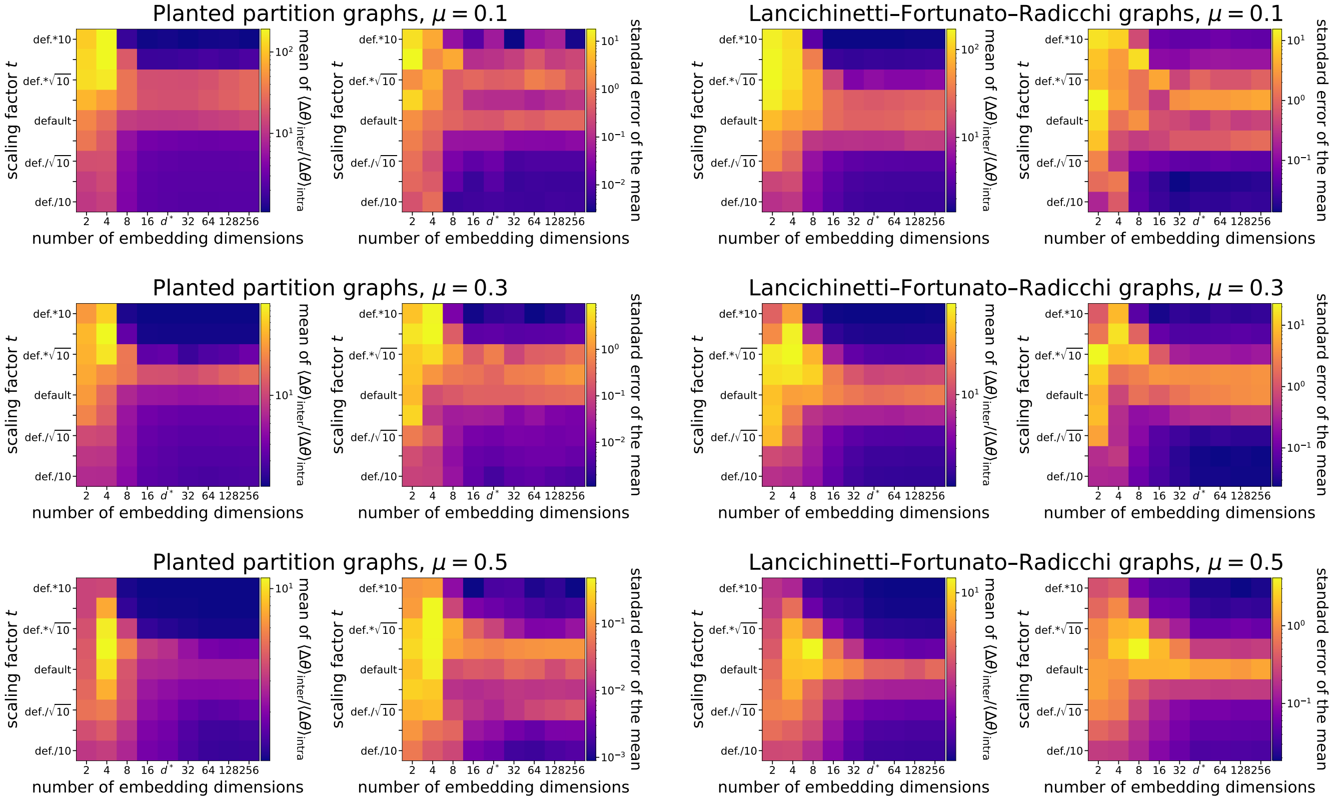

Figure S7. **The effect of the embedding parameters on the ratio between the average inter-community angular distance $\langle\Delta\theta\rangle_{\mathrm{inter}}$ and the average intra-community angular distance $\langle\Delta\theta\rangle_{\mathrm{intra}}$ at the final step when iterating node2vec with exponentialized link weights.** Each pair of subplots depicts the results for 10 realizations of a given type of synthetic networks: the left half of the figure refers to planted partition graphs (also examined in Fig. 3 of the main text), while the panels on the right deal with Lancichinetti–Fortunato–Radicchi networks (also examined in Fig. 4 of the main text). The IERW procedure was performed only once for each network. The default scaling factor was set according to our formula defined in Sect. S3.2, and d^* is the number of embedding dimensions chosen by our algorithm described in Sect. S3.1.

S4 Improvement of the weight thresholding performance with the iteration of the embedding

In the main text, Figs. 3 and 4 demonstrated that the angular separation between the communities planted in PP and LFR graphs can be significantly increased through the iteration of an embedding. Besides, Fig. 5 of the main text showed that by changing the link weights, iterated embeddings in many cases separate the inter-community links from the intra-community edges so well that they can make even a simple weight thresholding able to reveal the planted community structures with a quality comparable to that of well-known network community detection methods. Supplementing these results, Fig. S8 confirms that the iteration of the embeddings is indeed necessary for achieving convincing community detection performances with the weight thresholding procedure described in the Methods section of the main text, as this rather crude approach in general performs very poorly when applied after only a single embedding.

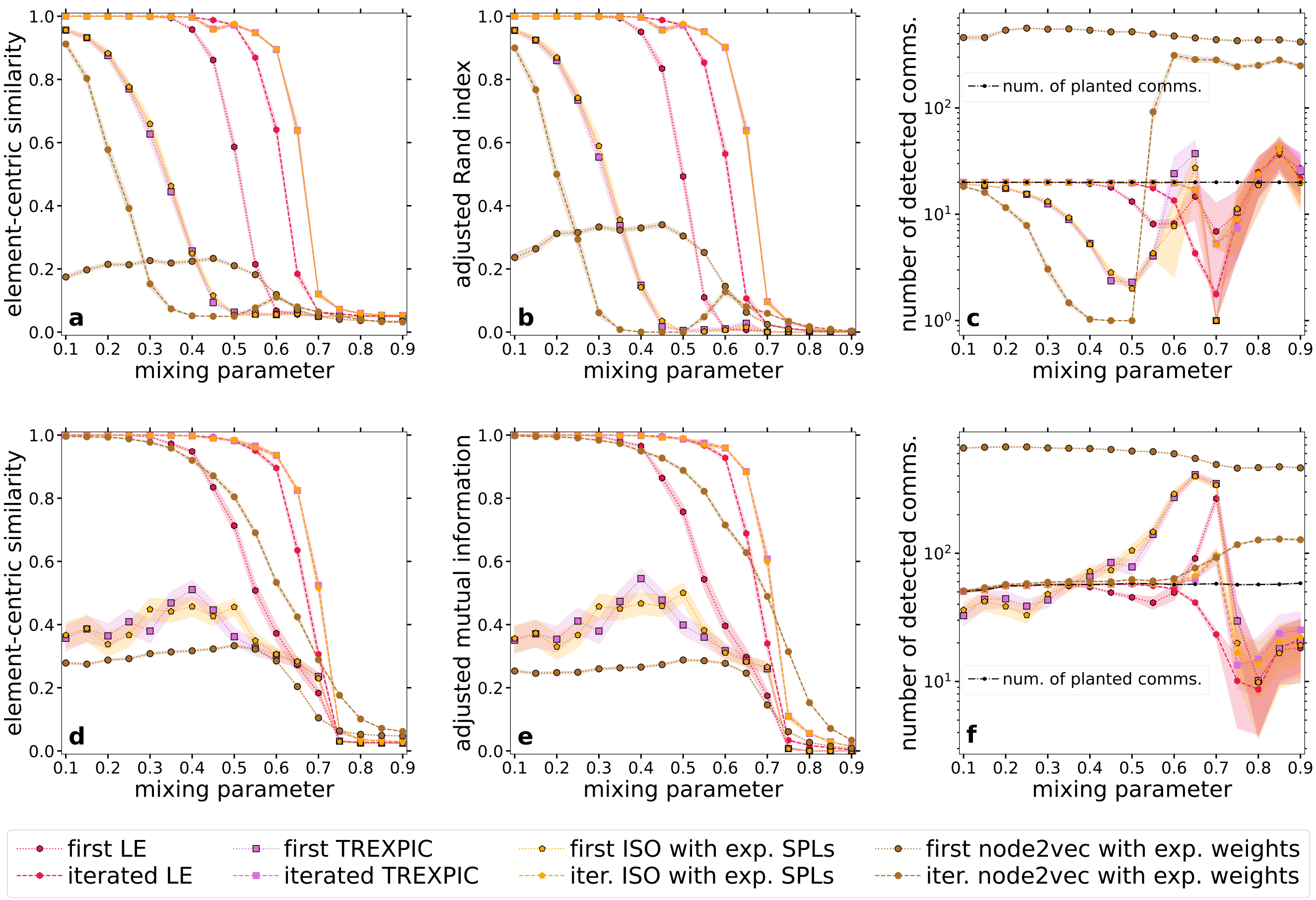

Figure S8. Community detection performance of weight thresholding with one single embedding and with the iterated embedding in the synthetic networks also examined in Fig. 5 of the main text. The upper row (panels **a**, **b** and **c**) deals with the networks generated by the planted partition model, and the lower row (panels **d**, **e** and **f**) refers to the graphs yielded by the Lancichinetti–Fortunato–Radicchi benchmark. The dotted lines present the results obtained from a single embedding with Laplacian Eigenmaps (LE, red hexagons), TRansformation of EXponential shortest Path lengths to hyperbolIC measures (TREXPIC, purple squares), Isomap (ISO) with exponentialized shortest path lengths (orange pentagons) and node2vec with exponentialized link weights (brown circles), whereas the dashed lines show the results achieved by iterating the above embeddings. Each data point corresponds to the average over 100 networks of the given parameter settings and the error bars show the standard error of the mean. We performed the community detection with all the methods only once for each network.

S5 Improving traditional community detection methods with matrix factorization embedding methods

While Fig. 6 in the main text demonstrated how IERW can be used with node2vec to improve the performance of traditional community detection methods, this section presents similar applications of iterating matrix factorization embedding techniques. Section S5.1 exemplifies through Louvain[20,21], asynchronous label propagation[22,23] and Infomap[24,25] that well-known network community detection methods can effectively utilize the link weights obtained from IERW with LE, TREXPIC and ISO. Besides, Sect. S5.2 illustrates through the example of Hierarchical Density-Based Spatial Clustering of Applications with Noise (HDBSCAN)[26–28] that standard spatial clustering methods can also benefit from iterating the LE, the TREXPIC and the ISO embedding methods.

S5.1 Enhancing network community detection using IERW with LE, TREXPIC and ISO

As pointed out in the main text, while IERW with LE, TREXPIC and ISO embeddings yields distance-like link weights (where higher values indicate weaker connections or more distant relations), the Louvain[20,21], the asynchronous label propagation[22,23] and the Infomap[24,25] community detection methods expect proximity-like link weights (where higher values indicate more intensive, stronger or closer relations). Therefore, before the application of Louvain, asynchronous label propagation or Infomap, one has to perform a conversion of the link weights provided by IERW with LE, TREXPIC and ISO. As mentioned in Eq. (4) of the main text, this can be done e.g. by following a similar practice to the one suggested in Ref.[4] and using the conversion formula

$$\tilde{w}_{ij} = \frac{1}{w_0 + w_{ij}}, \tag{S16}$$

where $w_0 > 0$ is a tunable parameter. As w_0 decreases, the links with small distance-like weights w_{ij} will be inputted to the traditional network community detection methods as stronger and stronger connections, while larger values of w_0 yield less difference between the resulting $\tilde{w}_{ij}$ proximity-like link weights of a network.

Figures S9 and S10 demonstrate in the case of the LE embedding algorithm how the performance of the different network community detection methods depend on the w_0 parameter of the distance-proximity conversion formula given by Eq. (S16). According to the similarity scores obtained at the four different settings of w_0 on the same planted partition[17] (PP) and Lancichinetti–Fortunato–Radicchi[18] (LFR) networks, Louvain — which, due to the resolution limit of the modularity[29], has a tendency of failing in detecting small communities — needs relatively small values of w_0 that put rather strong emphasis on the distance-like weights (i.e., cosine distances) that are close to 0. For the PP networks at $w_0 = 10.0$ (Fig. S9j) and for the LFR networks at $w_0 = 1.0$ and $w_0 = 10.0$ (Fig. S10g,j), the embedding-based link weights become unable to lead Louvain to the correct solution. Thus, for the Louvain method a relatively small w_0 seems to be the optimal choice, e.g. $w_0 = 0.1$.

On the other hand, it seems that small values of w_0 may highlight links of nearly 0 distance-like weights so much that they make Infomap — which has a much stronger tendency to find small communities than Louvain — returning smaller groups of nodes within the planted communities instead of the whole planted communities. In such cases (Fig. S10c,f,i), the first embedding provides more help than the iterated embedding for Infomap. This may be attributed to the emergence of some even denser patches within the relatively dense point clouds created by the first embedding from the planted communities, which is a natural consequence of the fact that due to the inhomogeneities of the connection structure, the spatial contraction of the individual communities is inhomogeneous during the embedding iteration. Therefore, the best solution for Infomap seems to be setting w_0 to a relatively large value of 10, avoiding thereby the identification of the smaller subclusters of the planted groups as separate communities and making Infomap focusing on the larger point clouds that correspond to the planted communities. As an intermediate case between Louvain and Infomap, when using asynchronous label propagation, the iterative embedding has the largest advantage compared to a single (i.e., the first) embedding at $w_0 = 1.0$ (Figs. S9h and S10h).

After making our choices regarding the w_0 parameter of Eq. (S16) based on the examples shown in Figs. S9 and S10, we tested the considered network community detection methods on the weighted graphs obtained from IERW with LE, TREXPIC and the exponentialized version of ISO, using $w_0 = 0.1$ in the case of Louvain, $w_0 = 1.0$ in the case of asynchronous label propagation and $w_0 = 10.0$ in the case of Infomap. Similarly to Fig. 6a–f in the main text that showed that IERW with node2vec can be used for facilitating these traditional network community detection methods, Figs. S11–S13 demonstrate that the link weights obtained from LE, TREXPIC or ISO in our IERW framework are capable of improving the performance of Louvain, asynchronous label propagation and Infomap.

S5.2 Enhancing HDBSCAN using IERW with LE, TREXPIC and ISO

In the main text, Fig. 6g–h demonstrated that the iteration of node2vec embeddings (using exponential link weights) provides a significant boost to the spatial clustering method Hierarchical Density-Based Spatial Clustering of Applications with Noise (HDBSCAN)[26–28]. Here, Fig. S14 investigates the performance of HDBSCAN in relation to the examined matrix factorization

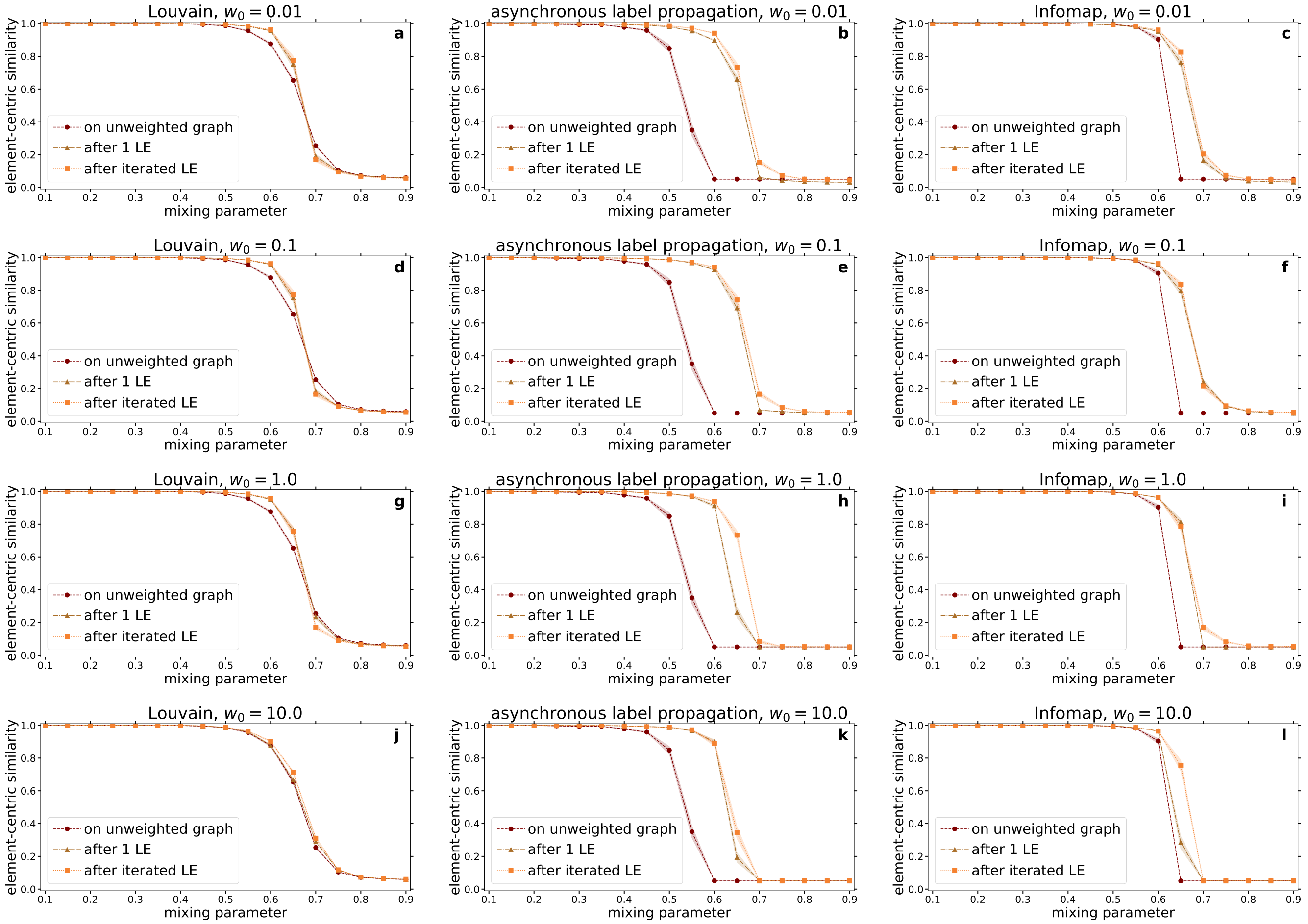

Figure S9. Performance of usual network community detection methods without embedding, with one Laplacian Eigenmaps embedding and with iterated Laplacian Eigenmaps embedding in planted partition networks, using the embeddings with different w_0 values. Each row of panels corresponds to a given setting of the w_0 parameter of Eq. (S16), and the different columns of panels refer to different community detection methods: Louvain, asynchronous label propagation and Infomap. We performed the community detection with all the examined methods only once for each network. Each displayed data point corresponds to a result averaged over 100 networks, and the error bars depict the standard error of the mean.

embedding methods. Since HDBSCAN accepts any type of distance matrix as an input, we tested it using not only the usual pairwise geometric distances (measured directly in the embedding space, disregarding the connectedness of the nodes) but also distances measured along the network links. Namely, when running HDBSCAN on an embedding, we used the matrix of pairwise cosine distances $1-\cos(\Delta\theta)$ between the embedded nodes, and when running HDBSCAN on a graph, we inputted the matrix of shortest path lengths (SPLs) of the (possibly weighted) graph. According to our IERW procedure, the link weights were defined for all three embedding methods by the cosine distances between the connected nodes in the embedding (see Eqs. (S2), (S7) and (S10)). As it is shown by Fig. S14, HDBSCAN was not able to find the planted communities based on the SPLs measured on the original, unweighted graphs (where each hop has the same contribution to the SPL and moving from one community to another can take only a single hop) even when the mixing between the communities was small. However, with the application of embedding-based link weights, the inter- and intra-community distances measured along the network links become much more distinguishable, raising the performance of the graph-based HDBSCAN to a level comparable to that of the embedding-based HDBSCAN. While the embedding-based HDBSCAN still seems to perform better after a single embedding, when the embedding is iterated, the communities become so strongly highlighted both in the spatial node arrangement and in the graph structure that it enables HDBSCAN to identify the planted communities at a similar high quality based on both types of inputs.

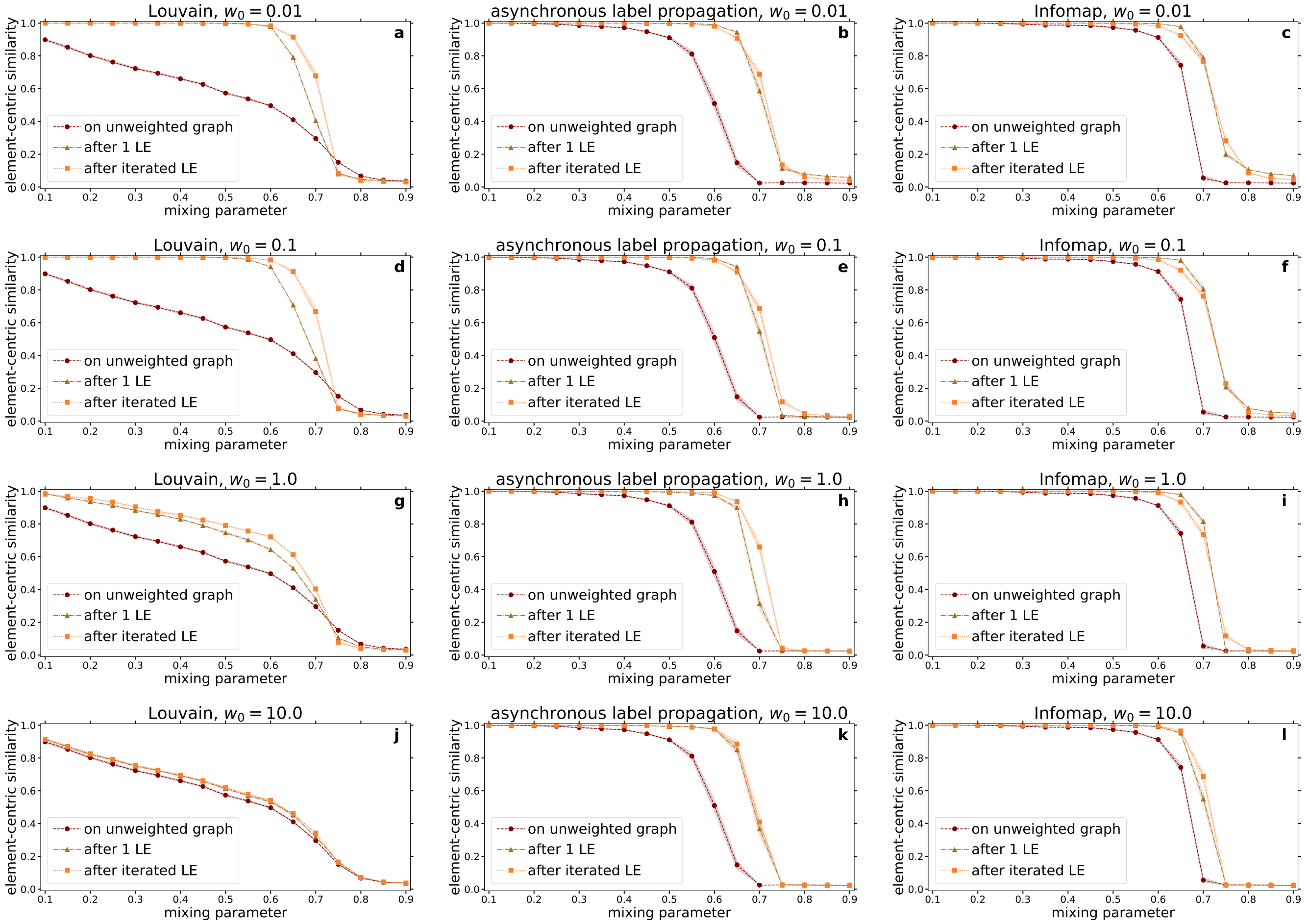

Figure S10. Performance of usual network community detection methods without embedding, with one Laplacian Eigenmaps embedding and with iterated Laplacian Eigenmaps embedding in Lancichinetti–Fortunato–Radicchi networks, using the embeddings with different w_0 values. Each row of panels corresponds to a given setting of the w_0 parameter of Eq. (S16), and the different columns of panels refer to different community detection methods: Louvain, asynchronous label propagation and Infomap. We performed the community detection with all the examined methods only once for each network. Each displayed data point corresponds to a result averaged over 100 networks, and the error bars depict the standard error of the mean.

S6 Exploring a hierarchical community structure with IERW

This section demonstrates how IERW works on a network that has hierarchical community structure, highligthing through this example once again the importance of using the proper number of embedding dimensions. The example graph was generated according to the hierarchical stochastic block model[30–32], distributing 1000 nodes in 10 larger blocks and 3 smaller clusters within each larger block. We set the number of vertices per each large block to 100, the number of vertices in the 3 smaller clusters within each larger block to 30, 30 and 40, the Bernoulli rates for the 3 smaller clusters within each larger block to

$$\begin{bmatrix} 0.30 & 0.10 & 0.15 \\ 0.10 & 0.35 & 0.20 \\ 0.15 & 0.20 & 0.40 \end{bmatrix}, \tag{S17}$$

and the Bernoulli rate of connections between vertices belonging to different large blocks to 0.1.

Figure S15 shows the ratio between the average inter-community angular distance $\langle\Delta\theta\rangle_{\text{inter}}$ and the average intra-community angular distance $\langle\Delta\theta\rangle_{\text{intra}}$ as a function of the number of IERW iterations performed for the above-described graph. In Fig. S15a, the 10 larger blocks are considered as the communities, while Fig. S15b depicts the angular separation of the 30 smaller blocks. Just like in the case of the synthetic networks of simpler community structures, the $\langle\Delta\theta\rangle_{\text{inter}}/\langle\Delta\theta\rangle_{\text{intra}}$ ratio tends to increase over the iterations for both levels of the community structure. As expected, IERW at $d = 9$ fits the planted community structure

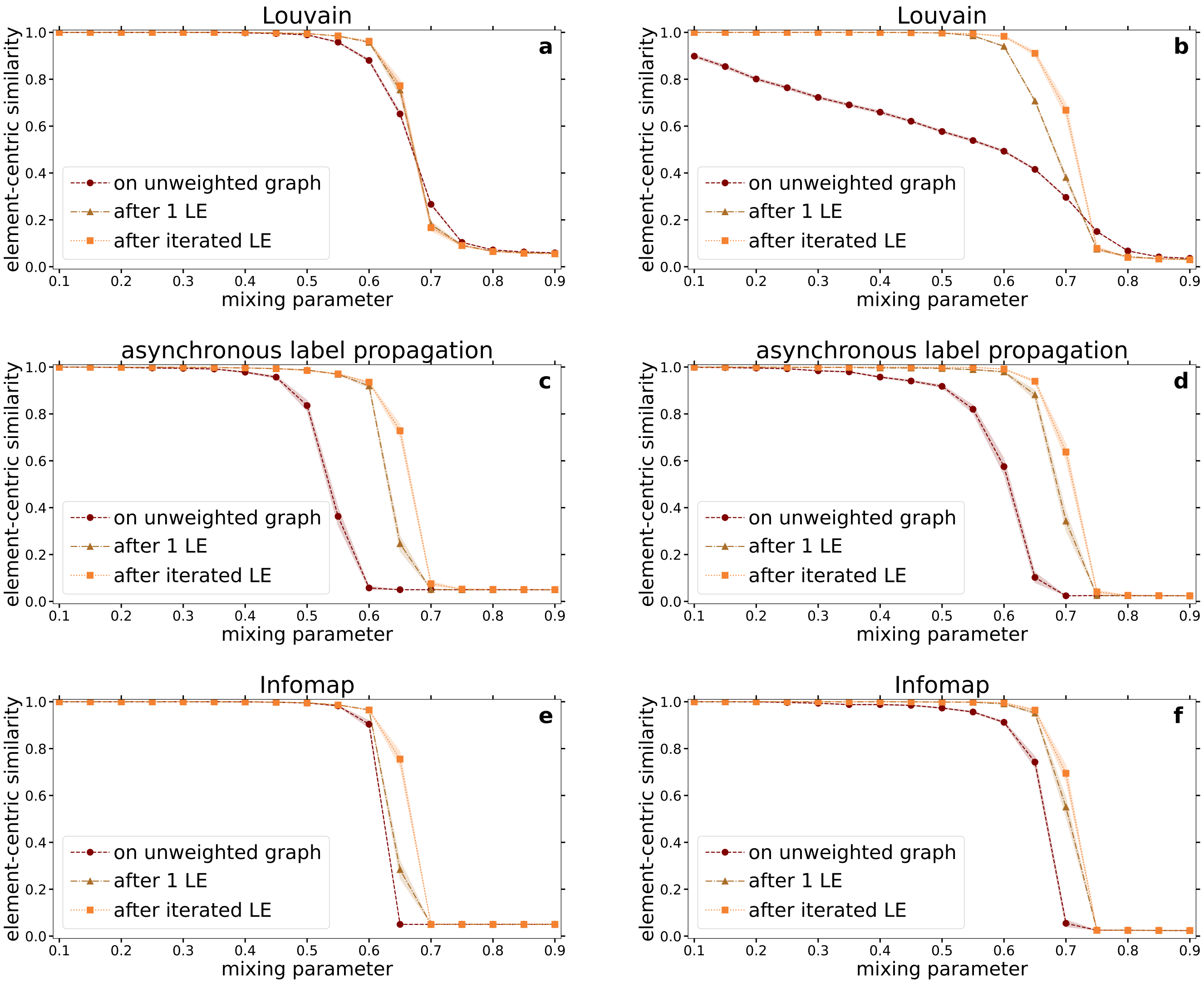

Figure S11. Performance of usual network community detection methods on the weighted networks derived by IERW using Laplacian Eigenmaps and the "optimal" w_0 values. Each row of panels corresponds to a different community detection method, and the left column refers to networks generated by the planted partition model, while the right one to networks generated by the Lancichinetti–Fortunato–Radicchi benchmark. We used here Eq. (S16) with the "optimal" settings of w_0 determined based on Figs. S9 and S10, namely with $w_0 = 0.1$ in the case of Louvain (panels a and b), $w_0 = 1.0$ in the case of asynchronous label propagation (panels c and d) and $w_0 = 10.0$ in the case of Infomap (panels e and f). We performed the community detection with all the methods only once for each network. Each displayed data point corresponds to a result averaged over 100 networks, and the error bars depict the standard error of the mean.

of 10 blocks better than $d = 29$, which is generally more suitable for separating the 30 smaller blocks from each other.

Similar tendencies can be observed with regard to community detection performances: Table S1 shows that IERW with LE, TREXPIC or ISO yields better results for both levels of the community structure when setting the number of embedding dimensions d in accordance with the considered number of planted communities C. Meanwhile, the performance of IERW with node2vec does not show an obvious preference towards the $d = C - 1$ setting, underpinning our network-independent choice of $d = 64$ in this case. Besides, Table S1 clearly indicates that IERW can significantly contribute to the identification of communities even in this more complicated, hierarchical case, achieving higher similarity scores than e.g. Louvain even with the very simple weight thresholding, but also being able to boost the performance of the considered traditional community detection methods through link weighting.

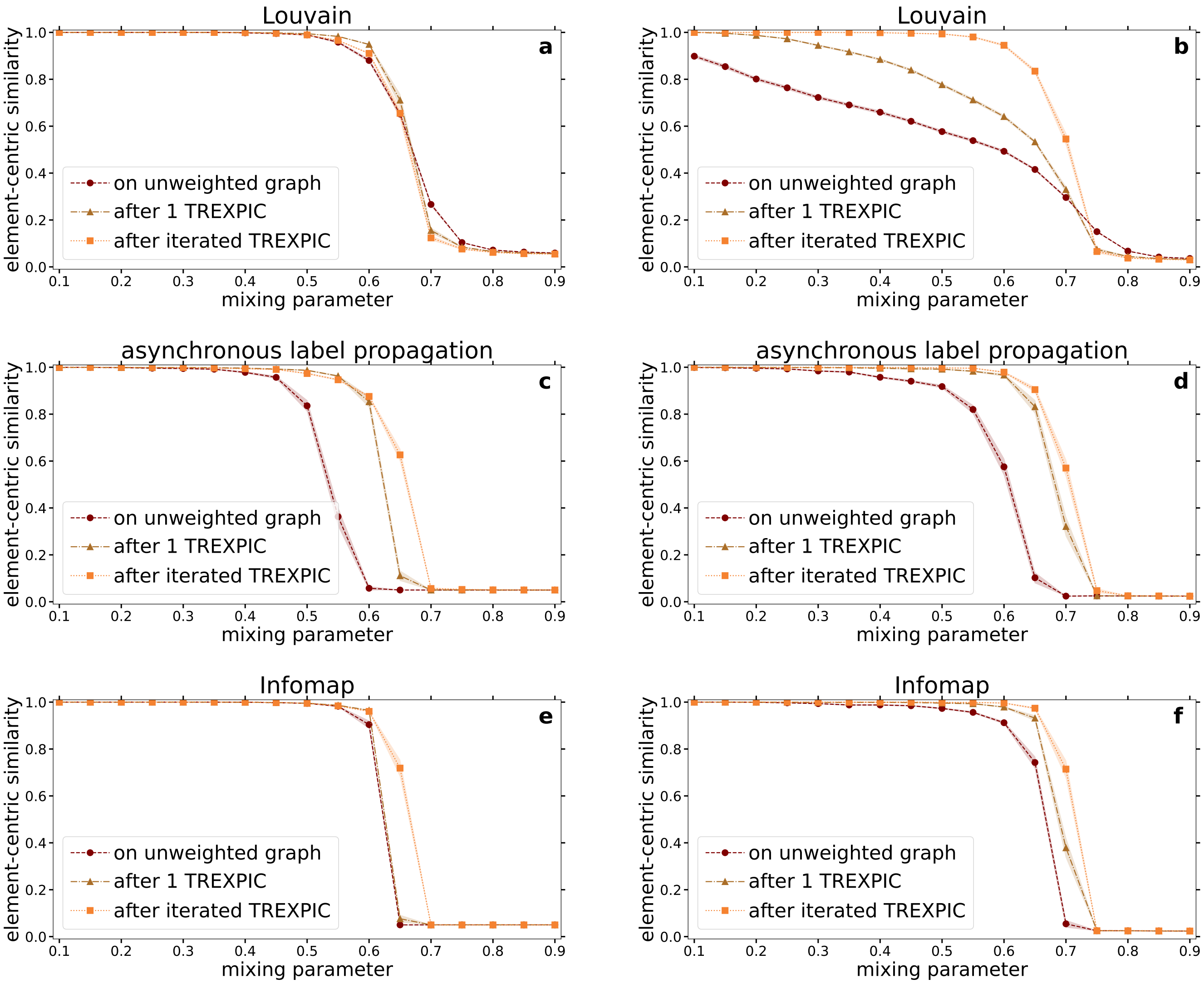

Figure S12. Performance of usual network community detection methods on the weighted networks derived by IERW using TREXPIC and the "optimal" w_0 values. The different rows of panels correspond to different community detection methods (named in the panel titles), and the left column of panels (i.e. panels **a**, **c** and **e**) refers to the networks generated by the planted partition model, while the right column of panels (i.e. panels **b**, **d** and **f**) deals with the networks obtained from the Lancichinetti–Fortunato–Radicchi benchmark. We used Eq. (S16) with the setting $w_0 = 0.1$ in the case of Louvain (panels a and b), $w_0 = 1.0$ in the case of asynchronous label propagation (panels c and d) and $w_0 = 10.0$ in the case of Infomap (panels e and f). We performed the community detection with all the examined methods only once for each network. Each displayed data point corresponds to a result averaged over 100 networks, and the error bars depict the standard error of the mean.

S7 Complexity and runtime analysis

The complexity of IERW depends on the chosen embedding algorithm, the dimension of the embedding, d and the number of iterations (i.e., embeddings and reweightings), k. In general, one reweighting step of the algorithm has a complexity $\mathcal{O}(E)$, where E denotes the number of links, which in sparse networks can be considered to be equivalent to $\mathcal{O}(N)$. For the scenarios considered in the main paper ($\ln(N) < d$), in the case of IERW with LE and TREXPIC, the complexity of a single embedding is $\mathcal{O}((d+1)\cdot N^2)$, yielding a total complexity of $\mathcal{O}(k\cdot((d+1)\cdot N^2+E))$, which is equivalent to $\mathcal{O}(k\cdot(d+1)\cdot N^2)$ in sparse graphs. Similarly, the complexity of a single embedding with ISO is $\mathcal{O}(d\cdot N^2)$, yielding a complexity of $\mathcal{O}(k\cdot d\cdot N^2)$ when used in IERW on sparse graphs. Besides, when using IERW with node2vec, the complexity of a single embedding is $\mathcal{O}(E+d\cdot\omega^2\cdot N)$ (where ω denotes the window length), resulting in a total complexity of $\mathcal{O}(k\cdot(E+d\cdot\omega^2\cdot N+E))$, which in

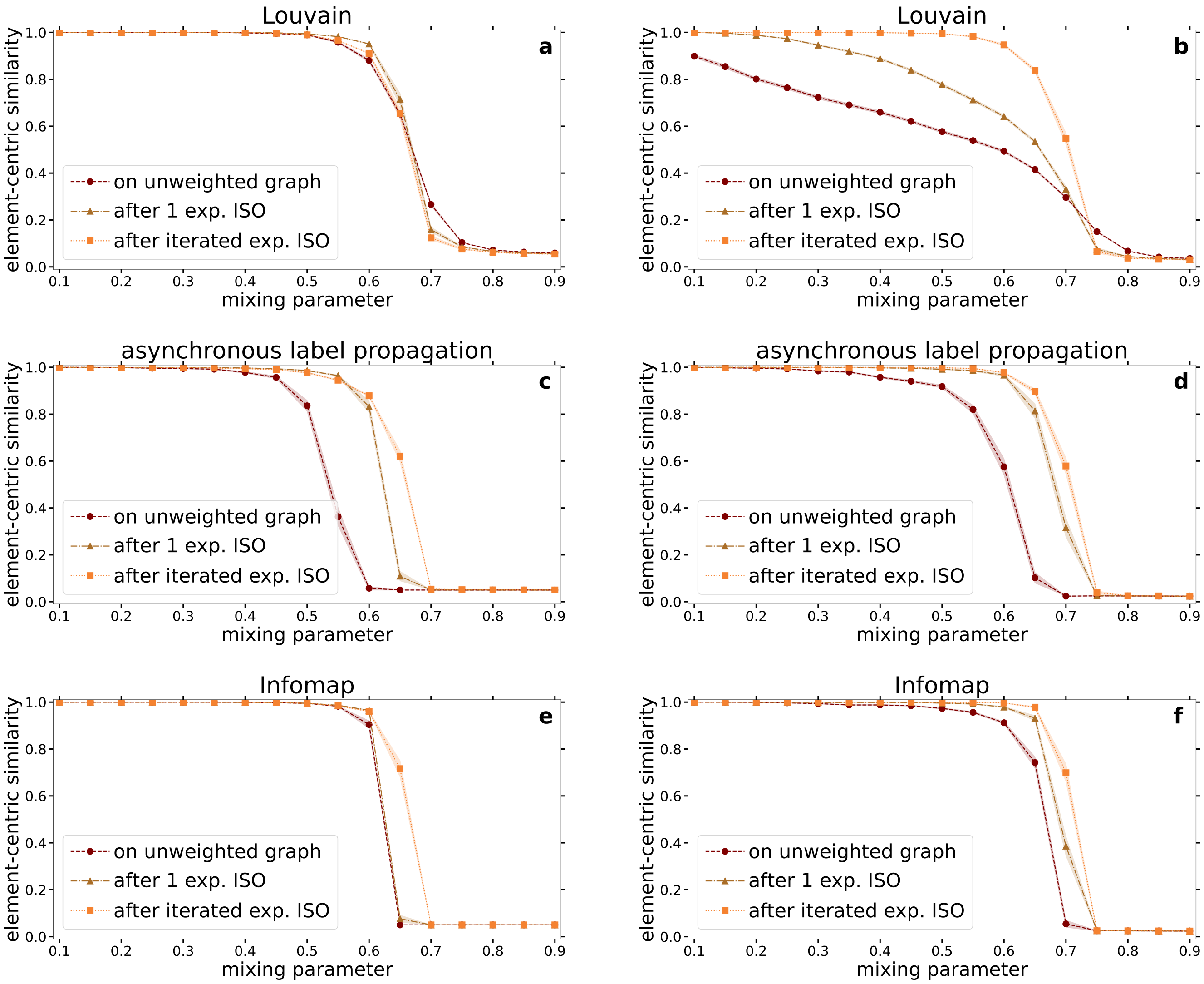

Figure S13. Performance of usual network community detection methods on the weighted networks derived by IERW using Isomap with exponentialized shortest path lengths and the "optimal" w_0 values. The different rows of panels correspond to different community detection methods (named in the panel titles), and the left column of panels (i.e. panels **a**, **c** and **e**) refers to the networks generated by the planted partition model, while the right column of panels (i.e. panels **b**, **d** and **f**) deals with the networks obtained from the Lancichinetti–Fortunato–Radicchi benchmark. We used Eq. (S16) with the setting $w_0 = 0.1$ in the case of Louvain (panels a and b), $w_0 = 1.0$ in the case of asynchronous label propagation (panels c and d) and $w_0 = 10.0$ in the case of Infomap (panels e and f). We performed the community detection with all the examined methods only once for each network. Each displayed data point corresponds to a result averaged over 100 networks, and the error bars depict the standard error of the mean.

sparse networks becomes equivalent to $\mathcal{O}(k \cdot d \cdot \omega^2 \cdot N)$.

In comparison, for the traditional community finding algorithms considered in the paper, the complexity of the Louvain algorithm is $\mathcal{O}(N \cdot \ln(N))$, the complexity of asynchronous label propagation is $\mathcal{O}(N + k \cdot E)$ (where k is the number of iterations over the network links during the repeated update of the labels), whereas the complexity of Infomap is $\mathcal{O}(r \cdot E \cdot \ln(N))$, where r is the number of restarts. Accordingly, the complexity of IERW is lower compared to Louvain or Infomap and is at level with asynchronous label propagation when embedding the networks using node2vec, whereas the complexity of IERW based on LE, TREXPIC or ISO is higher than for all the listed traditional community finding methods.

Although the complexity analysis shows what we can expect for the behaviour of the runtime when $N \to \infty$, in practice, for smaller networks additional factors not appearing in the big-O notation can also seriously affect which method runs faster or slower. Therefore, we have also carried out an empirical runtime analysis. The tests were run on a computer equipped with

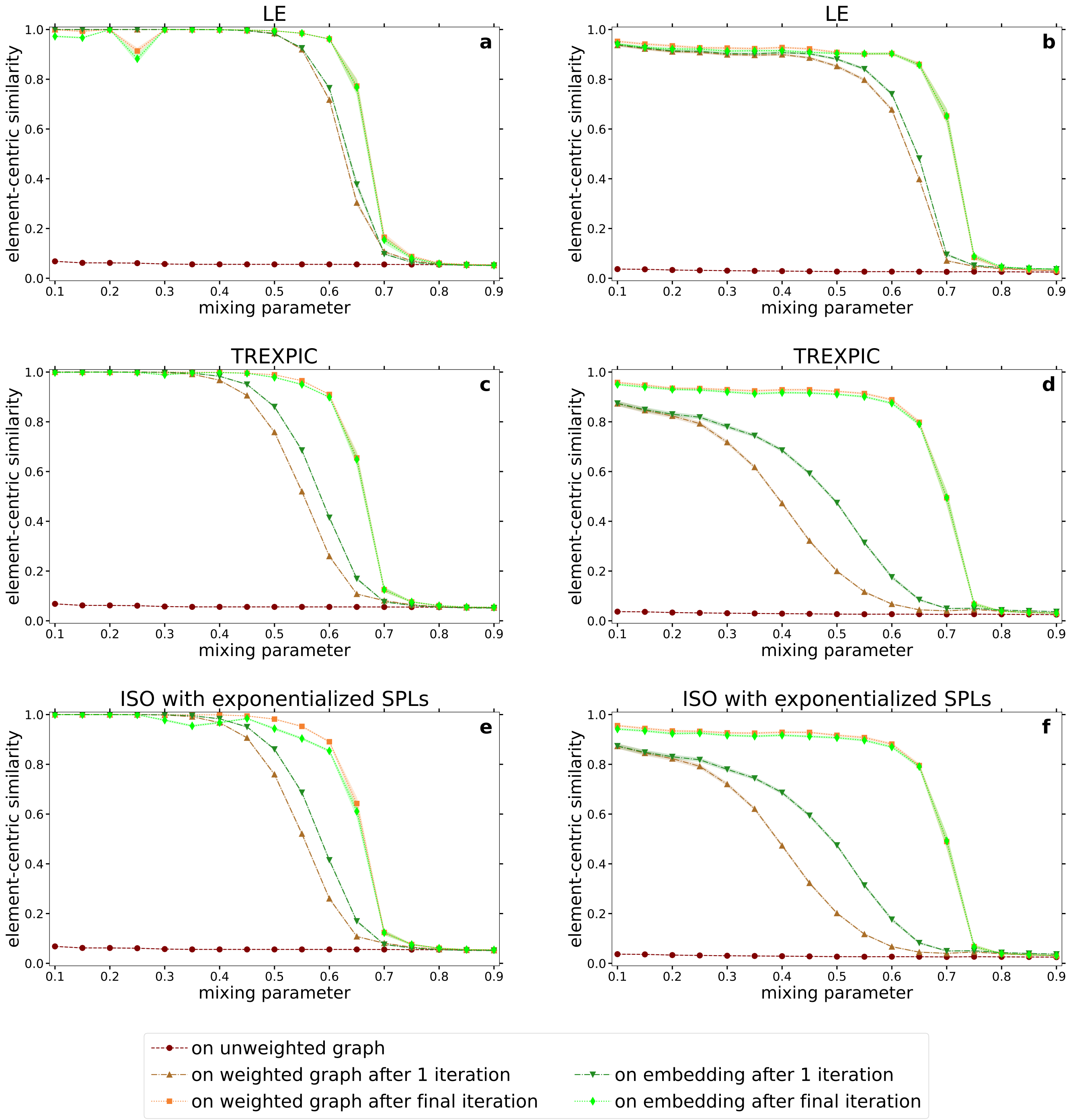

Figure S14. Performance of HDBSCAN on the weighted networks and embeddings derived by IERW using embeddings based on matrix factorization. The different rows of panels correspond to different embedding algorithms (named in the panel titles), and the left column of panels (i.e. panels **a**, **c** and **e**) refers to networks generated by the planted partition model, while the right column of panels (i.e. panels **b**, **d** and **f**) deals with the networks obtained from the Lancichinetti–Fortunato–Radicchi benchmark. In each panel, in the case of the two green curves, we used HDBSCAN on the matrix of cosine distances measured between the embedded nodes, while in the other three cases, we inputted into HDBSCAN the shortest path lengths measured along the network links. We performed all variants of the clustering only once for each network. Each displayed data point corresponds to a result averaged over 100 networks, and the error bars depict the standard error of the mean.

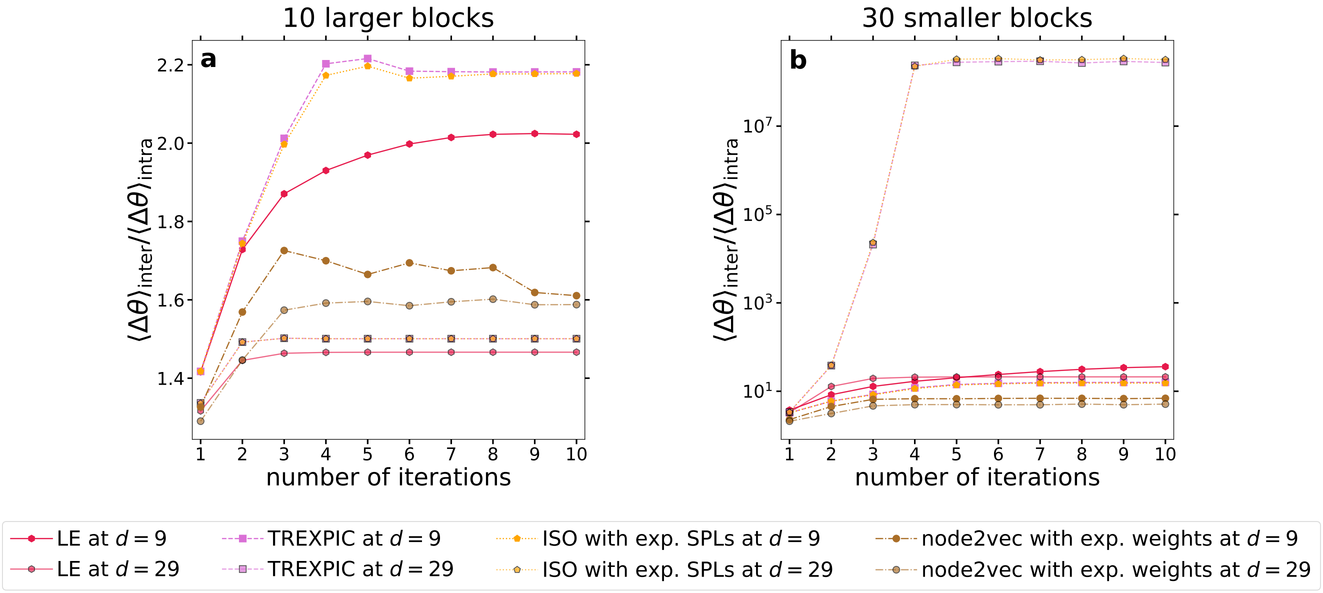

Figure S15. Angular separation of communities during IERW on the SBM graph having a two-level community structure of 10 larger and 30 smaller blocks. The different panels correspond to the different levels of the planted community structure: panel **a** to the 10 larger and panel **b** to the 30 smaller blocks. Each panel depicts the ratio between the average angular distance of all possible node pairs in different blocks and in the same block as a function of the number of IERW iterations for LE (red hexagons), TREXPIC (purple squares), ISO with exponentialized shortest path lengths (orange pentagons) and node2vec with exponentialized link weights (brown circles).

Intel(R) Xeon(R) Gold 6240 CPU 2.60 GHz and 16 GB RAM, running Linux Debian 4.19.160-2. Figure S16 shows the results of a measurement regarding the CPU time of the three traditional community detection methods that we used as reference points and 5 iterations of IERW with the studied four embedding methods. In this measurement, we examined planted partition networks of different sizes. The expected average degree $\bar{\kappa}$ was set to 20, and the mixing parameter μ was 0.3 in each network. To eliminate the differences that would emerge between the different embedding methods due to the application of different numbers of embedding dimensions, we planted $C = 65$ communities in each test graph and run all the embeddings using the setting $d = 64$. We used seven different settings of the number of nodes per community ($m = 15, 30, 75, 150, 300, 750, 1500$), and thereby seven settings of the total number of nodes in the graphs ($N = 975, 1950, 4875, 9750, 19500, 48750, 97500$). Note that in the case of TREXPIC and ISO, the testing was stopped at $N = 19500$ due to the relatively large memory and CPU time usage of these methods.

According to Fig. S16, the empirical runtime of IERW in our experiments was at least an order of magnitude larger compared to the runtime of the traditional community finding methods used in this work. Although that seems somewhat discouraging, still, as indicated by the curves, IERW can be run on networks with roughly 100,000 nodes under less than an hour. Furthermore, as it is shown by our results, the application of IERW offers considerable benefit in the community detection quality, making it worth to use IERW as a pre-processing in community finding.

References

1. Belkin, M. & Niyogi, P. Laplacian eigenmaps and spectral techniques for embedding and clustering. In Dietterich, T., Becker, S. & Ghahramani, Z. (eds.) *Advances in Neural Information Processing Systems*, vol. 14 (MIT Press, 2001).
2. Alanis-Lobato, G., Mier, P. & Andrade-Navarro, M. Efficient embedding of complex networks to hyperbolic space via their laplacian. *Sci. Rep.* **6**, 301082, DOI: 10.1038/srep30108 (2016).
3. Alanis-Lobato, G., Mier, P. & Andrade-Navarro, M. A. Manifold learning and maximum likelihood estimation for hyperbolic network embedding. *Appl. Netw. Sci.* **1**, 10, DOI: 10.1007/s41109-016-0013-0 (2016).
4. Muscoloni, A., Thomas, J. M., Ciucci, S., Bianconi, G. & Cannistraci, C. V. Machine learning meets complex networks via coalescent embedding in the hyperbolic space. *Nat. Commun.* **8**, 1615, DOI: 10.1038/s41467-017-01825-5 (2017).
5. García-Pérez, G., Allard, A., Serrano, M. Á. & Boguñá, M. Mercator: uncovering faithful hyperbolic embeddings of complex networks. *New J. Phys.* **21**, 123033, DOI: 10.1088/1367-2630/ab57d2 (2019).

Table S1. Element-centric similarities achieved by different community detection approaches on the SBM graph having a two-level community structure of 10 larger and 30 smaller blocks. As a reference, the first three rows show the similarity scores achieved by traditional community detection methods on the original, unweighted graph. Below that, the performance of weight thresholding, Louvain, Infomap and asynchronous label propagation is listed when utilizing the link weights obtained from the final iteration of IERW with LE, TREXPIC, ISO with exponentialized shortest path lengths and node2vec with exponentialized link weights. Lastly, the bottom of the table indicates the quality of the communities found by HDBSCAN on the final embedding provided by IERW using the studied four embedding methods. The community detection was performed with all the methods only once in each setting.

Community detection method	search for 10 blocks at $d = 9$	search for 10 blocks at $d = 29$	search for 30 blocks at $d = 9$	search for 30 blocks at $d = 29$
Louvain on unweighted graph	0.462	0.462	0.443	0.443
Infomap on unweighted graph	0.100	0.100	0.034	0.034
async. label prop. on unweighted graph	0.100	0.100	0.034	0.034
weight threshold after IERW with LE	0.342	0.340	0.286	1.0
weight threshold after IERW with TREXPIC	0.510	0.340	0.323	1.0
weight threshold after IERW with ISO	0.510	0.340	0.323	1.0
weight threshold after IERW with node2vec	0.520	0.520	0.700	0.592
Louvain after IERW with LE	0.370	0.340	0.469	1.0
Louvain after IERW with TREXPIC	0.513	0.340	0.324	1.0
Louvain after IERW with ISO	0.510	0.340	0.323	1.0
Louvain after IERW with node2vec	0.520	0.568	0.700	0.664
Infomap after IERW with LE	0.100	0.100	0.034	0.034
Infomap after IERW with TREXPIC	0.100	0.100	0.034	0.034
Infomap after IERW with ISO	0.100	0.100	0.034	0.034
Infomap after IERW with node2vec	0.520	0.502	0.700	0.700
async. label prop. after IERW with LE	0.472	0.3489	0.562	0.94
async. label prop. after IERW with TREXPIC	0.429	0.336	0.871	0.966
async. label prop. after IERW with ISO	0.420	0.358	0.735	0.870
async. label prop. after IERW with node2vec	0.394	0.351	0.610	0.034
HDBSCAN after IERW with LE	0.402	0.340	0.605	1.0
HDBSCAN after IERW with TREXPIC	0.521	0.340	0.351	1.0
HDBSCAN after IERW with ISO	0.520	0.340	0.380	1.0
HDBSCAN after IERW with node2vec	0.376	0.338	0.940	0.993

6. Jankowski, R., Allard, A., Boguñá, M. & Serrano, M. Á. D-mercator: multidimensional hyperbolic embedding of real networks (2023). Preprint at arXiv:2304.06580 [physics.soc-ph].

7. Kovács, B. & Palla, G. Model-independent embedding of directed networks into euclidean and hyperbolic spaces. *Commun. Phys.* **6**, 28, DOI: 10.1038/s42005-023-01143-x (2023).

8. Krioukov, D., Papadopoulos, F., Kitsak, M., Vahdat, A. & Boguñá, M. Hyperbolic geometry of complex networks. *Phys. Rev. E* **82**, 036106, DOI: 10.1103/PhysRevE.82.036106 (2010).

9. Ortiz, E., García-Pérez, G. & Serrano, M. Á. Geometric detection of hierarchical backbones in real networks. *Phys. Rev. Res.* **2**, 033519, DOI: 10.1103/PhysRevResearch.2.033519 (2020).

10. Trusina, A., Maslov, S., Minnhagen, P. & Sneppen, K. Hierarchy measures in complex networks. *Phys. Rev. Lett.* **92**, 178702, DOI: 10.1103/PhysRevLett.92.178702 (2004).

11. Keller-Ressel, M. & Nargang, S. Hydra: a method for strain-minimizing hyperbolic embedding of network- and distance-based data. *J. Complex Networks* **8**, DOI: 10.1093/comnet/cnaa002 (2020).

12. Tenenbaum, J. B., de Silva, V. & Langford, J. C. A global geometric framework for nonlinear dimensionality reduction. *Science* **290**, 2319–2323, DOI: 10.1126/science.290.5500.2319 (2000).

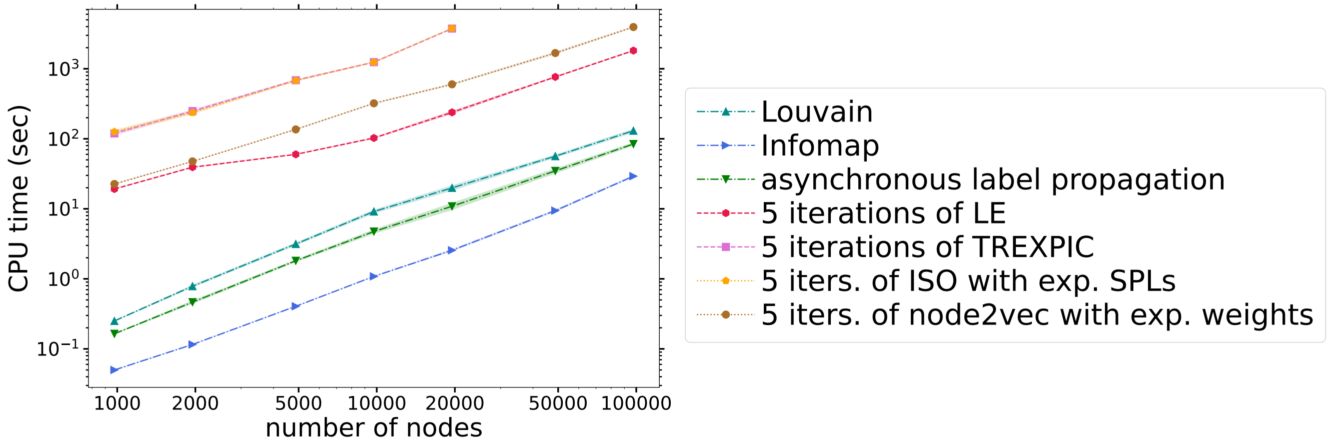

Figure S16. Runtime of IERW compared to the runtime of traditional community detection algorithms. At each network size, the measurement was performed on 10 different planted partition graphs. The displayed data points correspond to the averaged results and the error bars depict the standard error of the mean. We run all four embedding methods with the same setting of the number of embedding dimensions, namely $d = 64$. To make the comparison between the different embedding algorithms easier, we performed 5 iterations with each of them on each network.

13. Ou, M., Cui, P., Pei, J., Zhang, Z. & Zhu, W. Asymmetric transitivity preserving graph embedding. In *Proceedings of the 22nd ACM SIGKDD International Conference on Knowledge Discovery and Data Mining*, KDD '16, 1105–1114, DOI: 10.1145/2939672.2939751 (Association for Computing Machinery, New York, NY, USA, 2016).

14. Grover, A. & Leskovec, J. Node2vec: Scalable feature learning for networks. In *Proceedings of the 22nd ACM SIGKDD International Conference on Knowledge Discovery and Data Mining*, KDD '16, 855–864, DOI: 10.1145/2939672.2939754 (2016).

15. Mikolov, T., Chen, K., Corrado, G. & Dean, J. Efficient estimation of word representations in vector space (2013). Preprint at arXiv:1301.3781 [cs.CL].

16. Tandon, A. *et al.* Community detection in networks using graph embeddings. *Phys. Rev. E* **103**, 022316, DOI: 10.1103/PhysRevE.103.022316 (2021).

17. Condon, A. & Karp, R. M. Algorithms for graph partitioning on the planted partition model. *Random Struct. & Algorithms* **18**, 116–140, DOI: 10.1002/1098-2418(200103)18:2<116::AID-RSA1001>3.0.CO;2-2 (2001).

18. Lancichinetti, A., Fortunato, S. & Radicchi, F. Benchmark graphs for testing community detection algorithms. *Phys. Rev. E* **78**, 046110, DOI: 10.1103/PhysRevE.78.046110 (2008).

19. Lee, J. R., Gharan, S. O. & Trevisan, L. Multiway spectral partitioning and higher-order cheeger inequalities. *J. ACM* **61**, DOI: 10.1145/2665063 (2014).

20. Blondel, V. D., Guillaume, J.-L., Lambiotte, R. & Lefebvre, E. Fast unfolding of communities in large networks. *J. Stat. Mech. Theory Exp.* **2008**, P10008, DOI: 10.1088/1742-5468/2008/10/p10008 (2008).

21. We used the Python implementation of the Louvain algorithm available at https://github.com/taynaud/python-louvain.

22. Raghavan, U. N., Albert, R. & Kumara, S. Near linear time algorithm to detect community structures in large-scale networks. *Phys. Rev. E* **76**, 036106, DOI: 10.1103/PhysRevE.76.036106 (2007).

23. We used the Python function 'asyn_lpa_communities', an implementation of the asynchronous label propagation algorithm available in the 'networkx.algorithms.community.label_propagation' package.

24. Rosvall, M. & Bergstrom, C. T. Multilevel compression of random walks on networks reveals hierarchical organization in large integrated systems. *PLOS ONE* **6**, 1–10, DOI: 10.1371/journal.pone.0018209 (2011).

25. We used the Python package for the Infomap algorithm available at https://pypi.org/project/infomap/.

26. McInnes, L. & Healy, J. Accelerated hierarchical density based clustering. In *2017 IEEE International Conference on Data Mining Workshops (ICDMW)*, 33–42, DOI: 10.1109/ICDMW.2017.12 (2017).

27. Campello, R. J. G. B., Moulavi, D. & Sander, J. Density-based clustering based on hierarchical density estimates. In Pei, J., Tseng, V. S., Cao, L., Motoda, H. & Xu, G. (eds.) *Advances in Knowledge Discovery and Data Mining*, 160–172 (Springer Berlin Heidelberg, Berlin, Heidelberg, 2013).

28. We used the Python package for the HDBSCAN algorithm available at https://pypi.org/project/hdbscan/.

29. Fortunato, S. & Barthélemy, M. Resolution limit in community detection. *Proc. Natl. Acad. Sci. USA* **104**, 36–41, DOI: 10.1073/pnas.0605965104 (2007).

30. Csardi, G. & Nepusz, T. The igraph software package for complex network research. *InterJournal* **Complex Systems**, 1695 (2006).

31. Csárdi, G. *et al. igraph: Network Analysis and Visualization in R*, DOI: 10.5281/zenodo.7682609 (2024). R package version 2.0.3.

32. We used the R function 'sample_hierarchical_sbm' available in the 'igraph' package at https://igraph.org/r/html/1.3.4/sample_hierarchical_sbm.html for generating graphs with hierarchical community structure.
